# Learning from the Messengers: Innate Sensing of Viruses and Cytokine Regulation of Immunity—Clues for Treatments and Vaccines

**DOI:** 10.3390/v5020470

**Published:** 2013-01-31

**Authors:** Jesper Melchjorsen

**Affiliations:** 1 Department of Infectious Diseases, Aarhus University Hospital, Skejby, Denmark; E-Mail: jesper.melchjorsen@ki.au.dk; Tel.: +45-784-52842; Fax: +45-784-52848; 2 Department of Clinical Medicine, Aarhus University, Aarhus, Denmark; 3 Egaa Gymnasium, Mejlbyvej 4, Egaa, Denmark

**Keywords:** virus, innate, PRR, inflammation, IFN, cytokine, therapy, ISG, immune-modulatory, antiviral, vaccine, human, TLR, RLR, DNA

## Abstract

Virus infections are a major global public health concern, and only via substantial knowledge of virus pathogenesis and antiviral immune responses can we develop and improve medical treatments, and preventive and therapeutic vaccines. Innate immunity and the shaping of efficient early immune responses are essential for control of viral infections. In order to trigger an efficient antiviral defense, the host senses the invading microbe via pattern recognition receptors (PRRs), recognizing distinct conserved pathogen-associated molecular patterns (PAMPs). The innate sensing of the invading virus results in intracellular signal transduction and subsequent production of interferons (IFNs) and proinflammatory cytokines. Cytokines, including IFNs and chemokines, are vital molecules of antiviral defense regulating cell activation, differentiation of cells, and, not least, exerting direct antiviral effects. Cytokines shape and modulate the immune response and IFNs are principle antiviral mediators initiating antiviral response through induction of antiviral proteins. In the present review, I describe and discuss the current knowledge on early virus–host interactions, focusing on early recognition of virus infection and the resulting expression of type I and type III IFNs, proinflammatory cytokines, and intracellular antiviral mediators. In addition, the review elucidates how targeted stimulation of innate sensors, such as toll-like receptors (TLRs) and intracellular RNA and DNA sensors, may be used therapeutically. Moreover, I present and discuss data showing how current antimicrobial therapies, including antibiotics and antiviral medication, may interfere with, or improve, immune response.

## Abbreviations

AIDS: Acquired immune deficiency syndrome
ADAR1: Adenosine deaminase acting on RNA 1
AdV: Adenovirus
AIM2: Absent in melanoma 2
AP-1: Activator protein 1
APOBEC3: Apolipoprotein B mRNA-editing, enzyme-catalytic, polypeptide-like 3
ASC: Apoptosis-associated speck-like protein containing a caspase recruitment domain
ATF2: Activating transcription factor 2
AZT: Azidothymidine
BMDC: Bone marrow-derived DC
CARD: Caspase recruitment domain
CCL5: CC chemokine ligand 5 (previously known as regulated upon activation, normal T cell expressed and secreted (RANTES))
CCR5: CC chemokine receptor 5
CMV: Cytomegalovirus
CLR: C-type lectin receptor
CXCL10: CXC chemokine ligand 10
CYPA: Cyclophilin A
DAI: DNA-dependent activator of IFN-regulatory factors
DAMP: Danger-associated molecular pattern
DC: Dendritic cell
DC-SIGN: Dendritic Cell-Specific Intercellular adhesion molecule-3-Grabbing Non-integrin
DDX41: DEAD (Asp-Glu-Ala-Asp) box polypeptide 41
DHX9: DEAD/H (Asp-Glu-Ala-Asp/His) box polypeptide 9
Ds: Double-stranded
EBV: Epstein Barr virus
E.Coli: Escherichia coli
EMCV: Encephalo myocarditis virus
ER: Endoplasmic reticulum
ERK: Extracellular signal-regulated kinase
Flu: Influenza virus
GAS: IFN-γ-activated site
GM-CSF: Granulocyte macrophage colony-stimulating factor
HBV: Hepatitis B virus
HBsAg: Hepatitis B surface antigen
HCV: Hepatitis C virus
HDV: Hepatitis delta virus
HGF: Hepatocyte growth factor
HIV: Human immunodeficiency virus
HMGB1: High mobility group box-1
HPV: Human papilloma virus
HSP: Heat shock protein
HSV: Herpes simplex virus
ICP: Infected cell protein
IFIT1: Interferon-induced protein with tetratricopeptide repeats 1
IFI16: IFN-gamma-inducible protein 16
IFN: Interferon
IKK: Inhibitor of nuclear factor κb kinase
iNOS: Inducible nitric oxide synthetase
IRAK: IL-1R-associted kinase
IRF: Interferon regulatory factor
ISRE: Interferon-sensitive response element
JAK: Janus kinase
JNK: Jun N-terminal kinase
KSHV: Kaposi’s sarcoma-associated herpesvirus
LPS: Lipopolysaccharide
LRRFIP1: Leucine-rich repeat flightless-interacting protein 1
LTR: Long terminal repeat
MAPK: Mitogen-activated protein kinase
MAVS: Mitochondrial antiviral signaling protein
MDA5: Melanoma differentiation-associated gene 5
MDP: Muramyl dipeptide
MEF: Mouse embryonic fibroblasts
MHC: Major histocompatibility complex
MPL: Monophosphoryl lipid A
MyD88: Myeloid differentiation protein 88
NF-κB: Nuclear factor-κB
NLR: NOD-like receptor
NLRP3: NACHT, LRR and PYD domain-containing protein 3
CNS: Central nervous system
NO: Nitric oxide
NOD: Nucleotide-binding oligomerization domain
OAS: 2’-5’ oligoadenylate synthetase
ODN: Oligodeoxynucleotides
PAMP: Pathogen-associated molecular pattern
PBMC: Peripheral blood mononuclear cells
pDC: Plasmacytoid dendritic cells
PKR: Protein kinase R
PRR: Pathogen recognition receptor
PYHIN: Pyrin and HIN domain-containing protein
RLR: RIG-like receptor
RIG-I: Retinoic acid inducible gene I
RSV: Respiratory syncytial virus
RT: Reverse transcriptase
SAMHD1: SAM domain and HD domain-containing protein 1
SNP: Single-nucleotide polymorphism
STAT: Signal transducer and activator of transcription
STING: Stimulator of IFN genes
TBK1: TANK-binding kinase 1
TDF: Tenofovir disproxyl fumerate
TLR: Toll-like receptor
TNF-α: Tumor necrosis factor α
TRAF: TNF receptor-associated factor
TRIF: Toll/IL-1 receptor domain-containing adaptor inducing IFN-?
TRIM5α: Tripartite motif 5α
VAI: Adenoviral virus-associated type I
VSV: Vesicular stomatitis virus
VV: Vaccinia virus
VZV: Varicella zoster virus
WNV: West Nile virus

## 1. Introduction

Present and emerging viral infections pose an increasing burden to public health, and significant resources are used to limit the spread of virus infections. Major human viral pathogens include influenza A virus, causing annual epidemics and occasional pandemics; human immune deficiency virus 1 (HIV-1), the causative agent of acquired immune deficiency syndrome (AIDS); and herpes simplex virus (HSV), a significant cofactor of HIV infection and causative agent of genital and orofacial infections and viral encephalitis. Moreover, several viruses are the cause of life-long persistent infections and no protective vaccines have been developed. Overall, the lack of efficient vaccines for many viral infections, the suboptimal treatment for many viral infections, and the impact of viruses on human health and economy emphasize the need for an improved understanding of viruses’ natural history, including how the innate and adaptive immune responses may restrict virus infections, as well as modulate viral pathogenesis.

The innate immune system is the very first line of defense, and early recognition of invading pathogens is essential to initiate an antiviral response. However, the inflammatory response induced by virus recognition may also be detrimental to the host mediating immune-pathogenesis. Until a few years ago, the virus–host interactions responsible for initiation of antiviral responses were poorly characterized, but in recent years, knowledge on innate virus–host interactions have increased dramatically.

Cytokines are backbone molecules of the immune system, regulating growth, cell activation, differentiation of cells, attraction of cells to sites of infection, and exert direct antimicrobial effects. Especially interferons (IFNs) and proinflammatory cytokines, such as tumor necrosis factor α (TNF-α) and interleukin IL-12 (IL-12), play a major role in controlling viral infections. In addition, IFN-inducible proteins are very important in restricting virus infections [1,2].

This review summarizes the current knowledge on virus–host interactions. Furthermore, the review addresses innate stimulation as a mean of improving vaccine responses or as a direct antiviral mediator. Finally, the review discusses how current antimicrobial therapies may regulate innate responses and possibly interfere with or improve pathogen clearance.

## 2. Virus Activation of Pattern Recognition Receptors

**Figure 1:**
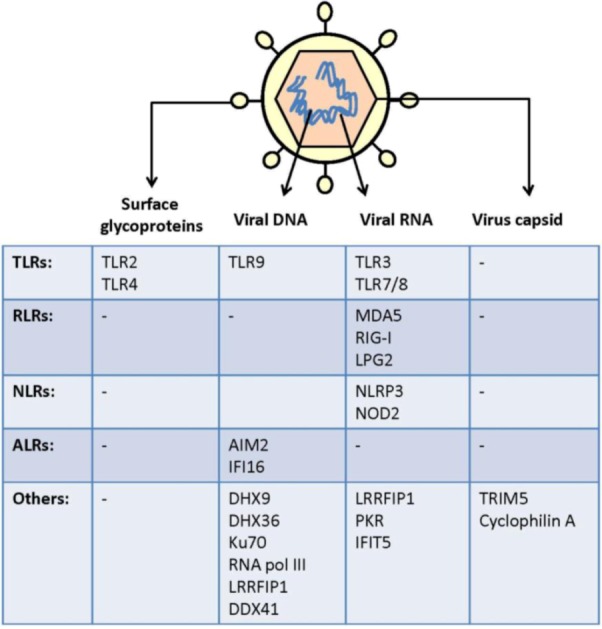
Viral PAMPs and cell PRRs. The viral particle constitutes of a viral genome of either RNA or DNA sensed by both membrane-associated receptors and receptors in the cytoplasm. DNA sensors include TLR9, AIM2, IFI16, DDX4, DHX9, DHX36, Ku70, and RNA pol III. RNA sensors include TLR3, TLR7/8, MDA5, RIG-I, NLRP3, NOD2, LRRFIP1, PKR, and IFIT1. The genome is surrounded by a capsid, which in the case of HIV is sensed by cyclophilin A (CYPA) and TRIM5. The outer surface of a major number of viruses consists of a lipid membrane with embedded glycoproteins. Virus surface structures are sensed by the cells *via* TLR2 and TLR4.

**Figure 2:**
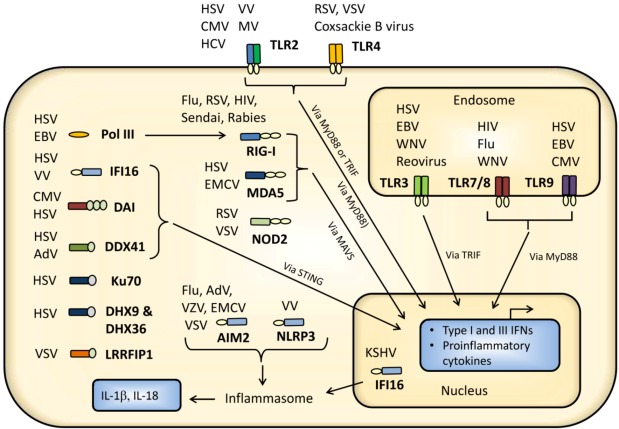
Viral sensors localized at membranes and in cytoplasm and nucleus. TLR2 and TLR4 located at the surface of the cell senses surface structures from a number of viruses, including HSV, CMV, VV, MV, RSV, and HCV. After internalization viral DNA genomes, typically from herpes viruses and VV, may be recognized by the DNA sensors IFI16, DAI, Ku70, AIM2, DDX41, RNA pol III, DHX9 or DHX36 localized in the cytoplasm or for IFI16 possibly also in the nucleus. Genomic DNA may also be recognized by TLR9 localized in endosomes. Viral genomic RNA or RNA structures’ accumulation during infection is recognized by the RLRs RIG-I or MDA5, the NLRs NOD2 or NLRP3 or the protein LRRFIP1. In addition, dsRNAs and ssRNAs localized in the endosomal compartments are recognized *via* TLR3 and TLR7/8, respectively. Signaling from TLRs proceed *via* the adaptor protein MyD88 (TLR2, TLR4, TLR7/8, and TLR9) and TRIF (TLR3 and TLR4). Signaling from DNA receptors is mediated *via* STING for at least the DNA receptors DAI, IFI16, and DDX41. Signaling from RIG-I and MDA5 proceeds *via* the signaling protein MAVS.

Invading viruses are recognized by several innate receptors located both at the cell surface and within the cells. The innate receptors generating the response are termed pattern recognition receptors (PRRs). In general, PRRs recognize conserved pathogen motifs termed pathogen-associated molecular patterns (PAMPs). Virus PAMPs include virus surface structures, virus genomic material, replication products, and capsids (Figure 1 and Table 1). Sensors of viral infections include toll-like receptors (TLRs), NOD-like receptors (NLRs), retinoic acid-inducible gene I (RIG-I)-like receptors (RLRs), and a number of cytoplasmic DNA receptors (Figure 1). Each virus may be sensed by several receptors and each sensor may sense several viruses (Figure 2). The following section will summarize and discuss the current knowledge on innate sensing of virus infections.

**Table 1:** Viruses and viral PAMPS.

Viruses	Genome	Family	PAMPs	Primary host (s)
**HSV**	dsDNA	Herpesviridae	Glycoproteins, dsRNA, Viral DNA	Human
**VZV**	dsDNA	Herpesviridae	Glycoproteins, dsRNA, Viral DNA	Human
**HCMV**	dsDNA	Herpesviridae	Glycoproteins, dsRNA, Viral DNA	Human
**EBV**	dsDNA	Herpesviridae	Glycoproteins, Viral DNA, RNAs	Human
**Vaccinia virus (VV)**	dsDNA	Poxviridae	Glycoproteins, Viral DNA, RNAs	Unknown
**Reovirus**	dsRNA	Reoviridae	dsRNA genome	Human
**Influenza A**	(-)ssRNA	Orthomyxoviridae	Viral 5’ppp ssRNA	Human, Pig, Fowl
**Measles virus**	(-)ssRNA	Paramyxoviridae	dsRNA, surface hemaglutinin	Human
**RSV**	(-)ssRNA	Paramyxovirus	dsRNA, ssRNA, proteins	Human
**Sendai virus**	(-)ssRNA	Paramyxoviridae	dsRNA, ssRNA virus genome	Mouse
**VSV**	(-)ssRNA	Rhabdoviridae	RNA	Many
**West Nile Virus**	(+)ssRNA	Flaviviridea	Genomic RNA	Human
**HCV**	(+)ssRNA	Flaviviridae	RNA, NS protein	Human
**Rhinovirus**	(+)ssRNA	Picornaviridea	RNA	Human
**Coxsackie virus**	(+)ssRNA	Picornaviridae	Virion, dsRNA	Human
**EMCV**	(+)ssRNA	Picornaviridae	dsRNA	Pig, rodent
**HIV**	ssRNA (RT)	Retroviridae	Genomic RNA, cDNA, capsid, glycoproteins	Human

### 2.1. Cell Surface Recognition of Virus

The major group of receptors recognizing a virus at the cell surface are the TLRs. Ten TLRs have been identified in humans with TLR1, TLR2, TLR4, and TLR6 primarily recognizing lipids and TLR3, TLR7, TLR8, and TLR9 recognizing nucleic acids [2]. TLRs are known to recognize multiple organisms, including a variety of viruses (Table 2). Best characterized are the receptors TLR2 and TLR4; TLR4 in complex with MD-2 primarily sensing extracellular gram-negative bacteria lipopolysaccharide (LPS) and TLR2 sensing bacterial lipopeptides and a number of fungal PAMPs. Interestingly, TLRs are capable of recognizing quite divergent motifs. As an example, TLR4 recognize LPS from gram negative bacteria, cell-derived danger-associated molecular patterns (DAMPs), as well as virus surface proteins [2]. The molecular basis of the broad range of PAMP structures recognized is not fully understood, but may partly rely on cellular distribution and involvement of PAMP-binding molecules, including MD-2 [3].

**Table 2:** Recognition of viruses by membrane-associated TLRs.

Receptor	Virus PAMP	Virus	References
**Cell surface TLRs**			
**TLR2**	Glycoproteins gH/gL	HSV	[4,5]
	Envelope glycoproteins	CMV	[6,7]
	Virion component, dUTase	EBV	[8,9]
	Not determined	VZV	[10]
	Hemagglutinin	Measles virus	[11]
	Core and nonstructural protein	HCV	[12]
	Not determined	VV	[13,14]
**TLR4**	Fusion protein	RSV	[15]
	Not determined	Coxsackie virus B	[16]
	Glycoprotein	VSV	[17]
**Endosomally located TLRs**			
**TLR3**	Virus-derived dsRNA	HSV	[18]
	EBER RNA	EBV	[19]
	Genomic dsRNA	Reovirus	[20]
	RNA	Influenza virus	[21,22,23]
	dsRNA	RSV	[24,25]
	dsRNA	HIV-1 vector	[26]
	dsRNA	Rhinovirus	[27,28]
	RNA	WNV	[29,30]
**TLR7/8**	Genomic ssRNA	HIV	[31,32,33]
	Genomic ssRNA	Influenza A	[34]
	Genomic ssRNA	Sendai	[35]
	Genomic ssRNA	Coxsackievirus B	[36]
	Genomic ssRNA	VSV	[34]
**TLR9**	Viral DNA	HSV	[37,38,39,40,41]
	Viral DNA	CMV	[42]
	Viral DNA	VZV	[43]
	Viral DNA	EBV	[44,45]
	Viral DNA	KSHV	[46]
	Viral DNA	VV	[47]
	Viral DNA	Adenovirus	[48,49]

#### 2.1.1. TLR2 and TLR4

The first report on virus recognition by TLRs was published in 2000, showing TLR4-mediated recognition of respiratory syncytial virus (RSV) in mice [15]. Later studies in humans have linked TLR4 polymorphisms to impaired resistance to RSV in high-risk infants [50]. In addition to RSV, the picornavirus Coxsackievirus B4 induces early cytokine production in pancreas cells TLR4 dependently [16], and vesicular stomatitis virus (VSV) glycoprotein G is recognized by TLR4 [17]. The *in vitro* data has been elaborated to *in vivo* findings showing that TLR4 deficient mice are more susceptible to high dose pulmonary vaccinia virus (VV) infections [51]. However, TLR4 may also mediate immune-pathogenesis during pulmonary virus infection, based on the findings that TLR4-mediated inflammation is detrimental during avian H5N1 influenza virus infection in mice [52].

TLR2 mediates recognition of measles virus hemagglutinin [11] and VV is recognized by TLR2 via an unknown PAMP [13]. TLR2 also senses cytomegalovirus (CMV) via virus envelope glycoproteins B and H activating nuclear factor κB (NF-κB) and cytokine production [6,7]. In addition to cytokine production, a group of inflammatory monocytes are capable of producing type I IFN after TLR2-mediated recognition of several DNA viruses, including VV [53]. It is noteworthy that a TLR2 single nucleotide polymorphism (SNP) has been associated with increased risk of CMV disease in liver transplant recipients, thus emphasizing TLR2s as an important mediator of antiviral defense against certain viruses [54]. Studies link TLR2 to recognition of herpes simplex virus (HSV) [4,55] and recent studies have identified HSV-1 glycoproteins gH/gL to mediate signaling via TLR2 [5]. Although TLR2 senses HSV infection in some cells, mouse studies suggest that TLR2 alone does not play an essential role in anti-HSV responses in mice [56]. Rather TLR2 may contribute to viral pathogenesis during HSV infection, evidenced by the finding that TLR2-deficient mice are resistant to viral encephalitis despite displaying similar viral loads compared with the wild type mice [4,56]. However, polymorphisms in TLR2 have been associated with increased viral shedding and lesion rate, suggesting a role for TLR2 during HSV-2 infection in humans [57]. TLR2 may also promote control of brain infections, since the presence of both functional TLR2 and TLR9 seems to be important for control of CNS infections in mice [58]. It should be noted that results gained in mice or mouse cells are not easily extrapolated to humans and human cells, as is exemplified by the finding that HSV-1 is recognized by TLR2 and TLR9 in murine DCs [40], whereas HSV-1 is recognized by human DCs independent of TLR2 [59].

In the context of virus infections, TLR4 and TLR2 may also recognizes damage-associated molecular patterns (DAMPs) released during infection. Both TLR2 and TLR4 have been associated with recognition of DAMPs released from necrotic infections, including heat shock proteins (HSPs), high mobility group box-1 (HMGB1) protein, and oxidized phospholipids [60], all of which may be released during virus infections [52,61,62]. Indicating a role of DAMPs in virus sensing, TLR4-deficient mice were found to be resistant to avian influenza-induced death during H5N1 avian influenza infection mediated by TLR4 recognition of cell-released oxidized phospholipids [52]. Future research will have to delineate whether some of the reported virus-induced immune responses mediated via TLR2 and TLR4 are due to recognition of DAMPs rather than direct recognition of the viruses, and thus further characterize the role of TLR2 and TLR4 during virus infection in humans.

#### 2.1.2. C-Type Lectins

C-type lectins (CLRs) consist of a large family of soluble and transmembrane proteins recognizing a large range of carbohydrate structures on pathogens. The CLR family includes dendritic cell-specific intercellular adhesion molecule-3-grabbing non-integrin (DC-SIGN) and mannose receptors both associated with innate recognition of viruses. DC-SIGN mediates rapid endocytosis of HIV-1 by DCs resulting in either destruction of the virus in the endosomes, survival and replication in the DCs, or intracellular transfer to T cells [63,64,65,66]. Mannose receptors mediate a similar HIV-1 transfer mechanism in macrophages [67]. In addition, DC-SIGN signaling events, in concert with TLR8 activation, promote HIV replication in DC [68]. Dengue virus, Ebola virus, and CMV are other viruses utilizing DC-SIGN as cellular receptor for infection of DCs [69,70,71]. Albeit, presently, no CTR-induced activation of NF-κB or IFN regulatory factor 3 (IRF3) and subsequent IFN production has been shown during virus infection; CLR may induce signaling via a number of kinases, including spleen tyrosin kinase (SYK) and Src kinase during bacterial and virus infections; thus, CLRs possibly shape innate responses during infection [72]. Collectively, CLRs together with TLR8 are important for productive HIV-1 infection of DCs, but CLRs may also participate in regulating immunity.

### 2.2. Endosomal Recognition of Viral RNA and DNA

TLR3, TLR7/8, and TLR9 are located in the endosomes and sense nucleic acids, such as viral genomes and accumulating viral RNAs [2]. Table 1 summarizes TLR-mediated recognition of viruses. 

#### 2.2.1. TLR3

TLR3 is expressed in many cells and senses short double-stranded (ds)RNA and triggers activation of NF-κB, mitogen-activated protein kinases (MAPKs), and IRF3 and subsequent IFN and cytokine responses [2,20]. TLR3 plays a direct role in recognition of virus infection, evidenced by the finding that genomic dsRNA from reovirus activates cytokine production [20]. In cell cultures, a number of productive virus infections, including HSV, Adenovirus, EMCV, and VSV infection result in accumulation of dsRNAs [76,77]. Correspondingly many virus infections are sensed by TLR3, including RSV (parainfluenza virus), rhinovirus (picornavirus), reovirus (reovirus), Epstein Barr Virus (EBV), and HSV-2 (both herpes viruses) [19,20,24,25,27,28,78]. Furthermore, antiviral and inflammatory response to influenza A virus and HIV-1 lentivirus vectors have been shown to be mediated via TLR3 [21,23,26]. Several studies have addressed TLR3’s role in virus infection using TLR3 deficient mice. Studies from mice have revealed that TLR3 is involved in the pulmonary antiviral response against RSV, but is not essential [25]. TLR3 deficiency, however, is linked with increased permissiveness to HSV-2 central nervous system (CNS) infection in mice [78]. In humans, HSV infection is also recognized by TLR3, evidenced by the finding that a deletion in TLR3 increases the risk of encephalitis in children and inhibits HSV-mediated stimulation of IFN-β, IFN-γ, and IL-6 in fibroblasts [18]. TLR3 may therefore be primarily important for combating virus infections in the CNS. However, the route of infection and type of virus may very well determine TLR3’s role during infection. An example is West Nile virus (WNV), where one study in mice showed TLR3-dependent neuronal protection and increased risk of encephalitis in TLR3-deficient mice [30], but another study showed increased survival in mice deficient in TLR3 when using another route of infection and another type of virus preparation [79]. TLR3-mediated immune-pathogenesis has also been observed for other viruses, such as influenza A virus and VV, evidenced by the findings that mice deficient in TLR3 produce less inflammatory cytokines and are more resistant to infections with influenza A virus or VV [22,79]. Finally, TLR3 may participate in generation of efficient adaptive response, since TLR3 stimulation helps virus-infected DC to cross-present antigens and generate efficient CD8+ T cells responses [80]. In conclusion, TLR3 is involved in innate recognition of very different classes of viruses and very important for restricting CNS infections caused by HSV in humans, whereas the role during WNV is less clear. Whether TLR3 deficiency also primes for encephalitis caused by other viruses, such as VZV or RSV, remains to be determined. 

#### 2.2.2. TLR7 and TLR8

TLR7 and TLR8 are highly expressed in plasmacytoid DCs (pDCs) and act as direct sensors of virus genomic material resulting in high levels of IFN-α expression [2]. TLR7 and TLR8 sense single-stranded uridine-rich (ss)RNA genomic material from a range of viruses. Influenza virus (orthomyxovirus), HIV-1 (retrovirus), VSV (rhabdovirus), sendai virus (paramyxovirus), and coxsackievirus B (picornavirus) are all sensed by TLR7 and TLR8 mediating IFN and cytokine responses [32,33,34,35,36]. Activation of human pDC and secretion of IFN-α has also been attributed TLR7 during dengue 2 virus infection [81]. In addition, TLR8-mediated recognition of AT-rich VV DNA genome has been proposed [82]. The authors found that pDC recognition of VV DNA was mediated by TLR8 and not TLR9 in murine pDCs [82]. The results are somewhat controversial and have been questioned by others in the field [83]. TLR7 may also sense infected cells exemplified by the finding that pDCs recognize HIV-1-infected T cells via a TLR7-dependent mechanism [84]. pDCs may also recognize HIV-1 directly, since genomic RNA from endocytosed HIV-1 activates pDCs via TLR7 [31]. Finally, TLR7 and TLR8 may also link to adaptive responses, evidenced by the findings that influenza A virus-infected DCs cross-prime antigens via an TLR7-dependent mechanism.

#### 2.2.3. TLR9

TLR9 is primarily expressed by pDCs in humans and a number of cells in mice [85]. pDCs are “professional IFN producers” with constitutive high levels of IRF7 and thus capable of rapidly producing high levels of IFN-α. TLR9 mediates sensing of unmethylated CpG motifs characteristic in virus and bacterial DNA genomes, including HSV-1 and HSV-2 genomic material, resulting in expression of IFN-α, IFN-λ, and a number of cytokines and chemokines [37,86,87,88]. The herpes viruses varicella zoster virus (VZV), CMV, and EBV have also been shown to mediate IFN-α production via TLR9 suggesting that all herpes viruses are sensed by TLR9 [42,43,44,45]. VV infection and adenovirus genomic DNA is also sensed by TLR9 [47,48,49]. Moreover, HIV-1 recognition via TLR9 has been proposed [31]. Several studies have addressed TLR9’s role in antiviral responses. TLR9 is essential for antiviral responses during VV infection in mice [47]. In humans, a study has shown that polymorphisms in TLR9 have been linked with rapid progression of HIV, suggesting that proviral DNA or cellular debris is important for immune function during HIV-1 infection. However, the finding has to be confirmed in other settings. In the case of HSV infections, present results indicate a nonessential role for TLR9 during most infections, albeit TLR9 may together with TLR2 play a role for control of HSV brain infection in mice [58].

Since TLR3, TLR7/8, and TLR9 are located in endosomes, the intriguing question remains: How do the virus RNAs and DNAs get in contact with the endosomal compartments, knowing that the viruses replicate in the nucleus or cytoplasm? At least three mechanisms may account for delivery of viral nucleic acids to the endosomes. First, RNAs or DNA present in the cytoplasm may be engulfed by an autophagosome, which subsequently fuse with the endosome [89]. In that way, pDC may utilize the cellular process termed autophagy, in which damaged organelles, proteins *etc*. are degraded in membrane-surrounded autophagosomes. The second option is direct endocytosis of virus particles or, alternatively, virus-infected cells, subsequently making the viral nucleic acids available for sensing by TLR3, TLR7/8, and TLR9. Finally, virus-infected cells may be sensed via cell–cell interaction. An example is a recent paper showing that pDCs recognize HIV-1-infected CD4+ T cells TLR7-dependently [84].

### 2.3. Cytoplasmic and Nuclear Recognition of Virus Infection

**Figure 3:**
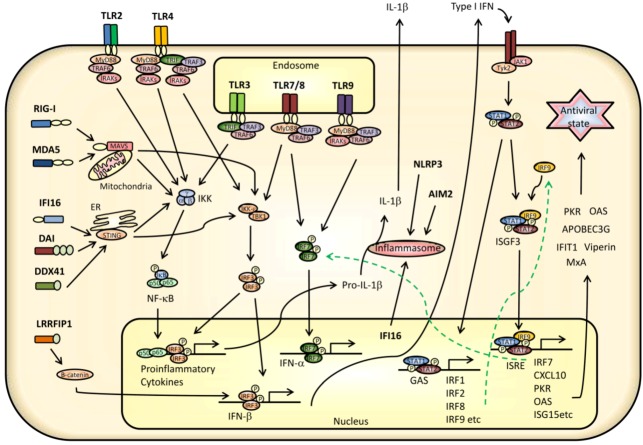
PRR activated signaling and antiviral responses during virus infection. An overview of signaling pathways triggering IFN and cytokine production and mediates an endogenous antiviral states after sensing of invading virus. TLR2, TLR4, TLR7/8, and TLR9 all signals *via* the adaptor molecule Myd88 and *via* TRAF6 and/or TRAF3 and IRAKs. TLR2 primarily mediates activation of NF-κB, whereas TLR3 and TLR4 also mediate activation of IRF3 *via* the adaptor molecule TRIF. TLR3 and TLR4 stimulation induce IFNβ production. TLR7/8 and TLR9 primarily activate IRF7 leading to immediate IFN-α expression, but also NF-κB activation leading to stimulation of proinflammatory cytokines. RNA sensors and DNA sensors lead to activation if both IRF3 and NF-κB thus regulating both IFN and cytokine production. DNA sensors IFI16, DAI, and DDX41 signals *via* STING located at the ER whereas the RNA sensors RIG-I and MDA5 signal *via* MAVS. Finally, the DNA sensors AIM2 and IIF16 and the RNA sensor NLRP3 participate in inflammasome formation and cleavage of pro-IL-1β to IL-1β. Secreted IFN binds to its receptor and activates JAK/STAT signaling pathways leading to enhanced IFN expression and induction of antiviral mediators, including OASs, PKR, IFIT1, APOBEC3, and viperin, as well as production of IRF7 inducing IFN-α responses and thus increase overall IFN secretion. The MAPK signaling pathway activated by several PRRs, including TLRs and RLRs, has been omitted in the figure. ER = endoplasmic reticulum.

The very recent years have brought us exiting knowledge on a variety of cytoplasmic and nuclear sensors of virus infections mediating IFN and cytokine response essential for successful antiviral responses (Figure 3). The receptors include: RNA sensors; Leucine-rich repeat flightless-interacting protein 1 (LRRFIP-1); RIG-like receptors (RLRs); and, NLRs. DNA receptors include DNA-dependent activator of IFN-regulatory factors (DAI), IFN-gamma-inducible protein 16 (IFI16), and DHX9/DHX36 (Table 3). The following section will focus on RNA and DNA receptors sensing virus infection in the cytoplasm.

#### 2.3.1. RIG-I-Like Receptors

The RLR family consist of the three DExD/H-box containing RNA helicases melanoma differentiation associated gene 5 (MDA5) (alternatively termed IFN-induced with helicase C domain 1, IFIH1), retinoic acid inducible gene I (RIG-I) (alternatively termed DDX57), and laboratory of genetics and physiology 2 (LGP2). The exact role of LGP2 in antiviral immune responses is debated, whereas both RIG-I and MDA5 have been linked to early recognition of virus infections mediating antiviral IFN and cytokine responses [35,77,91,92,95,101,105,134]. All three members of the RLR family bind RNA via a RNA-binding domain. RIG-I and MDA5 also encode N-terminal caspase recruitment domains (CARDs), which triggers signaling after binding of RNA to the receptors. Signaling is mediated via CARD–CARD interactions with the CARD-containing adaptor molecule mitochondrial antiviral signaling protein (MAVS) (also known as virus-induced signaling adaptor (VISA)), IFN-β promoter stimulator 1 (IPS-1), and CARD adaptor inducing IFN-β (cardif) [135,136,137,138]. Since LGP2 lacks a CARD domain, it does not mediate signaling, but LGP2 has been ascribed a function as both enhancer and inhibitor of RLR functions [139,140,141,142].

MDA5 (IFIH1) is a cytoplasmic receptor for long and higher structure dsRNAs [77,105]. MDA5 recognizes a diverse range of viruses, including VV (poxvirus), reovirus (reovirus), VSV (rhabdovirus), and the picornaviruses rhinovirus, and EMCV [27,28,92,97,98,105,143]. We and collaborators have shown that MDA5 recognizes HSV-1 in human primary macrophages [104]. In addition, Sendai virus-defective interfering particles, a byproduct of viral replication, induce IFN and DC activation via a MDA5-dependent mechanism [107]. Moreover, Sendai virus-induced IFN response is partially dependent on MDA5 in hepatocytes [134]. The innate response via MDA5 may be essential for virus-induced IFN responses and for clearance of the virus, as seen during EMCV infection [92]. MDA5-induced responses may, however, also be the cause of hyper responses and inflammation detrimental to the host, as seen during rhinovirus infections [27].

**Table 3:** Recognition of viruses by cytoplasmic and nuclear receptors.

Receptor	Virus PAMP	Virus	References
**Cytoplasmic RNA recognition**			
**RIG-I**	5´ppp viral RNA	Influenza A	[23,90,91,92]
	ssRNA and dsRNA	HIV	[93,94]
	Virus-encoded RNA	EBV	[95]
	dsRNA	Reovirus	[96,97]
	dsRNA	VV	[98]
	dsRNA	Measles virus	[99]
	RNA	RSV	[91,100]
	dsRNA	Sendai virus	[35,92,101]
	dsRNA	Human parainfluenza virus	[102]
	dsRNA	VSV	[92,101,103]
**MDA5**	dsRNA	HSV	[104]
	dsRNA	VV	[98,105]
	RNA	Reovirus	[97]
	dsRNA	Measles Virus	[99]
	dsRNA	Coxsackie B	[106]
	RNA	Sendai virus defective interfering particles	[107]
	dsRNA	Rhinovirus	[27,28]
	dsRNA	EMCV	[92]
**DDX60**	dsRNA	VSV	[108]
**DHX9**	dsRNA	Influenza A	[109]
	dsRNA	Reovirus	[109]
**DDX1/DDX21/DHX36**	dsRNA	Influenza A	[110]
**DDX1/DDX21/DHX36**	dsRNA	Reovirus	[110]
**NOD2**	ssRNA	RSV	[111]
	ssRNA	VSV	[111]
**NALP3**	M2 protein, RNA	Influenza	[112,113,114,115]
	Unknown	Sendai virus	[112]
	dsRNA	EMCV	[116]
	dsRNA	VSV	[116]
	Genomic DNA	Adenovirus	[117]
	unknown	VZV	[118]
**PKR**	dsRNA	HSV	[41,119]
	dsRNA	VV	[98]
**LRRFIP1**	RNA	VSV	[120]
**Cytoplasmic DNA recognition**			
**RNA pol III**	Genomic DNA	HSV	[121]
	Genomic DNA	EBV	[122]
**IFI16**	Genomic DNA	HSV	[123]
**DAI**	Genomic DNA	HSV	[124]
	Genomic DNA	CMV	[125]
**DHX9**	Genomic DNA	HSV	[126]
**DHX36**	Genomic DNA	HSV	[126]
**DDX41**	Genomic DNA	AdV	[127]
	Genomic DNA	HSV	[127]
**Ku70**	Genomic DNA	HSV	[128]
**AIM2**	Virus DNA	MCMV	[129]
	Virus DNA	VV	[129]
**Nuclear-located receptor for nucleic acids**			
**IFI16**	Virus genomic DNA	KSHV	[130]
**Miscellaneous**			
**Cyclophilin A**	Capsid	HIV	[131]
**TRIM5**	Capsid lattice	HIV	[132]
**NLRP3**	Membrane penetration	AdV	[75]
**IFIT1**	5’triphosphated viral RNA	Influenza A	[133]

RIG-I is sensor for shorter dsRNA and 5’ppp ssRNAs and mediates type I IFN responses in response to a number of viruses, including HIV-1, influenza A virus, EBV, RSV, reovirus, VV, Sendai virus, and human parainfluenza virus [35,90,91,91,92,93,94,95,97,98,101,102,134,143]. In addition to mediating IFN and proinflammatory cytokine production, RIG-I has also been shown to mediate activation of the inflammasome and thus IL-1β secretion in respond to the rhabdovirus VSV [103]. 

#### 2.3.2. Other DExD/H-Box Helicases

In addition to the RLRs, the DExD/H-box helicases DDX1, DDX21, DHX9, and DHX36 have been linked to recognition of cytoplasmic RNA and sensing of virus infections in myeloide DCs. DHX9 has been linked to sensing of dsRNA and mediate signaling via MAVS [109]. DDX1, DDX21, and DHX36 form a complex that recognizes dsRNA in the cytoplasm and senses infection with influenza A and reovirus [110]. The DDX1-DDX21-DHX36 complex was found to utilize the Toll/interleukin-1 receptor domain-containing adaptor inducing IFN-β (TRIF) pathway to activate expression of type I IFNs [110]. As described later, DHX9 and DHX36 also sense DNA in the cytoplasm of pDCs [126].

#### 2.3.3. NOD-Like Receptors

NLRP3 (Cryopyrin) recognizes several RNA viruses. Sendai virus, influenza virus and rotavirus dsRNAs are sensed by NLRP3 and NLRP3 mediates activation of the inflammasome in mice or mouse cells [112]. Moreover, VZV activates the NLRP3 inflammasome in human fibroblasts, THP-1 cells, and melanoma cells [118]. Moreover, and hepatitis C virus (HCV) activates the inflammasome and IL-1β secretion via NLRP3 in human hepatoma cells [144]. NLRP3 is also important for *in vivo* control of virus infections. In mice, NLRP3 mediates inflammation and production of cytokine and chemokines during influenza A infection and NLRP3 is important for restricting the infection, as evidenced by the finding that NLRP3-deficient mice show enhanced mortality compared to wt mice [113,115]. NLRP3 also mediates inflammasome activation and IL-1β release during respiratory RSV infection [145]. The role for NLRP3 is less clear for VSV activation of the inflammasome. One report shows that RIG-I binds to apoptosis-associated speck-like protein containing a caspase recruitment domain (ASC) in complex with caspase 1 independent of NLRP3 whereas another report claims NLRP3 to be a receptor for VSV RNA, independent of RIG-I [103,116]. Since NLRP3 has been shown to mediate inflammasome activation of the DNA virus VZV in human monocyte-like THP-1 cells and primary lung fibroblasts, it is possible that NLRP3 also recognizes DNA [118]. NLRP3 sensing of HIV-1 has been suggested based on finding that IL-1β is upregulated during HIV-1 infection of human DCs [146]. Further suggesting a role for NLRP3 for control of HIV-1 infection, polymorphisms in genes for NLRP3 and the inflammatory cytokine IL-1β are both associated with increased susceptibility to HIV-1 infection [147,148]. NLRs may also activate non-inflammasome, innate responses evidenced by the finding that NOD2 recognizes RSV ssRNAs and mediate innate responses, including IFN production [111].

#### 2.3.4. PKR

The cytoplasmic protein kinase R (PKR) is important for direct antiviral activity, but also plays a role in signaling. PKR is activated by dsRNA from viruses and is a component of MAPK and NF-κB signaling [164,165,166]. PKR is activated by 5-triphosphated RNAs with short stem loops, including HIV-1 TAR RNA [167,168]. PKR has been linked to viral innate responses. During HSV infection, type I IFN and inflammatory cytokines are produced PKR-dependently [41,119]. Whether the PKR dependent cytokine and IFN production relates to direct PKR signaling after activation by HSV-derived RNA remains to be determined. Furthermore, reovirus-induced IFN responses are regulated by PKR [97]. PKR may also attenuate virus–host responses via RLRs suggesting that the protein either sequester certain dsRNAs or interact with signaling. HSV utilizes PKR to inhibit type I IFN production via RIG-I [169]. It will be interesting to define whether other viruses suppressing IFN production, like HIV-1, actively utilizes PKR to inhibit RLR signaling. Moreover, PKR mediates autophagic degradation of HSV-1 [170], which possibly could mediate the transport of viral PAMPs to endosomally located TLR9 and/or enhanced presentation of endogenous viral antigens via major histocompatibility complex I (MHC I) [171]. Finally, PKR may be an important mediator of virus-induced apoptosis, evidenced by the finding that VV-derived RNA species and synthetic RNAs in the cytoplasm activate apoptosis dependent on PKR [98,172]. Thus, during virus infection, PKR may regulate innate PRR recognition, apoptosis, and signaling at several levels, as well as play a role for generation of adaptive responses.

#### 2.3.5. DNA Receptors

Viral and synthetic DNA has been known to induce IFN and ISG responses for more than a decade [149]. In recent years, several nuclear and cytoplasmic sensors of viral DNA have been identified, yet many questions still remain unanswered, including which intracellular DNA receptors are responsible for HIV-1-induced cytokine and caspase 1 and 3 responses observed in CD4+ T cells, and which DNA receptor mediates inflammasome activation in HSV-infected cells [129,150]. In the following section, the current knowledge on virus sensing by DNA receptors within the nucleus and cytoplasm of cells will be presented.

#### 2.3.6. DAI/ZBP-1

DAI (alternatively ZBP-1/DLM-1) recognizes HSV-1 genomic DNA in murine L929 cells and CMV genomic DNA in human fibroblasts mediating the expression of IFN-β [124,125]. In addition, DAI is upregulated during HSV-1 infection of astrocytes and glial cells and DAI facilitates production of inflammatory cytokines IL-6 and TNF-α during HSV-1 infection [151]. DAI may also contribute negatively to virus infection. One example is that DAI expression enhances HIV-1 replication via activation of NF-κB, which is known to enhance long terminal repeat (LTR)-driven expression [152]. Collectively, the present data suggest that DAI may both contribute to antiviral responses though the production IFNs from fibroblasts, but DAI may also potentially augment immuno-pathogenicity by mediating inflammation in the brain during infection. Moreover, DAI may enhance HIV-1 infection though stimulation of HIV-1 replication.

#### 2.3.7. Ku70

Ku70 is a DNA-binding protein with multiple functions, including telomere maintenance, DNA replication, antigen-receptor gene rearrangement, cell cycle control, and apoptosis [153]. One paper suggests that Ku70 is a sensor of cytosolic DNA and shows a role for Ku70 in production of IFN-λ during HSV-1 infection of HEK292 cells [128]. Whether the result can be extrapolated to others cells and other viruses remains to be determined. However, evidence suggests that Ku70 is not a general regulator for IFN-λ, since IFN-λ is induced via TLR3 in human fibroblasts and via MDA/MAVS in human primary macrophages [18,104].

#### 2.3.8. IFI16

The IFN-inducible protein IFI16 is a cytoplasmic and nuclear-located protein shown to recognize synthetic HSV-1 and VV DNA in cell cultures initiating innate IFN responses [123]. In contrast, adenovirus activates innate IRF3 signaling independent of IFI16 in murine RAW264.7 macrophage-like cells [154]. IFI16 may also mediate expression of the chemokine CCL3 in human primary macrophages during HSV-1 infection [74]. Moreover, IFI16 recognizes DNA from Kaposi sarcoma-associated herpes virus (KSHV), mediating activation of the inflammasome and IL-1β secretion from infected endothelial cells [130]. The involvement of IFI16 in activation of the inflammasome may be cell specific since IFI16 has been shown to suppress activation of caspase 1 by AIM2 and NLRP3 inflammasomes [155]. In addition, IFI16 is a restriction factor for herpes viruses, evidenced by the finding that knockdown of IFI16 augments CMV and HSV-1 replication in cell cultures [156,157]. In addition to the role in innate response, IFI16 may also affect adaptive responses, since activation of human DCs by cytoplasmic DNA is dependent on IFI16 [158]. Collectively, IFI16 is a multifunctional protein regulating very early innate response to infections with DNA viruses, regulating adaptive responses, and directly inhibiting viral replication. It remains to be determined whether IFI16 recognizes retroviruses encompassing a DNA step in the replication.

#### 2.3.9. RNA pol III

RNA polymerase III (RNA pol III) has been shown to sense cytoplasmic dsDNA and, via produced RNA, intermediates signal via RIG-I and MAVS. [121,122]. In murine bone marrow-derived DCs, RNA pol III mediates the accumulation of IFN-inducing small RNAs from adenovirus DNA via a mechanism partly dependent on MAVS [159]. In murine RAW267.4 cells, RNA pol III does not seem to affect the early innate response during adenovirus infection, but inhibition of RNA pol III attenuates the later innate response [154]. Sensing of HSV-1 by RNA pol III has also been proposed. Chiu *et al*. found that RNA pol III/RIG-I mediates IFN production in murine macrophage-like RAW264.7 cells during HSV-1 infection [121]. However, the results for HSV-1 are controversial, since others have shown that expression of IFN and cytokines proceeds via a RNA pol III-independent mechanism in both murine and human macrophages [104,123].

#### 2.3.10. DHX9, DHX36, DDX41, and DDX60

DEAD/H (Asp-Glu-Ala-Asp/His) box polypeptide 9 (DHX9), DDX60, DEAD (Asp-Glu-Ala-Asp) box polypeptide 41 (DDX41), and DHX36 belong to the DExD/H box helicase family. DHX9 and DHX36 have been shown to sense CpG-rich DNA in human pDCs [126] and as described earlier, DHX9 and DHX36 sense dsRNA in myeloid DCs [109,110]. In a human pDC line, DHX9 was found to mediate NF-κB activation via MyD88 and regulate TNF-α expression, whereas DHX36 was found to mediate IRF7 activation via MyD88 and mediate production of IFN-α [126]. DDX41 has recently been identified as a sensor of cytoplasmic DNA and a sensor of adenovirus and HSV-1 infection in murine bone marrow-derived DC and a myeloid DC line [127]. DDX41 was also found to mediate signaling via STING to promote expression of type I IFN in DCs [127]. Moreover, the authors found that knockdown of DDX41 inhibits secretion of IFN-β and IL-6 from human monocyte-like THP-1 cells during HSV-1 infection. DDX60 is involved in sensing of dsRNA and dsDNA [108]. Miyashita *et al*. found that the DDX60 mediates IFN-β and CXCL10 expression after transfection with dsRNAs and dsDNA and that DDX60 amplifies signaling from MDA5 and RIG-I. Furthermore, the authors found that CXCL10 and IFN-β expression was dependent on DDX60 during infection with HSV-1 and VSV in an endothelial cells line. It remains to be determined whether DDX60’s primary function is direct sensing of virus infections or rather regulate RLR signaling. Furthermore, it remains to be determined whether DHX9 and DHX36 sense other DNA viruses in cells different from the investigated pDCs. Finally, it remains to be determined whether DDX41 is only a DNA sensor in myeloid DCs.

#### 2.3.11. LRRFIP1

LRRFIP1 is a transcriptional regulator present both in the cytoplasm and in the nucleus. In addition, LRRFIP1 interacts with both RNA and DNA in the cytoplasm and mediates IFN-β production via a β-catenin pathway [120]. LRRFIP binds GC-rich regions, characteristic in herpes virus DNA genomes, making LRRFIP1 a likely candidate as mediator of IFN-β production. Presently, no studies have shown LRRFIP1 recognition of HSV. In addition to sensing cytoplasmic DNA and RNA, LRRFIP1 regulates TLR pathway signaling [160]. Thus LRRFIP1’s primary role during virus infections may be regulating signaling from other PRRs.

#### 2.3.12. AIM2

AIM2 is an IFN-inducible, DNA-binding protein belonging to the pyrin and HIN domain-containing protein family (PYHIN family). AIM2 is part of an ASC inflammasome and mediates activation of caspase 1 and cleavage of pro-IL-1β and pro-IL-18 to mature IL-1β and IL-18 [161,162]. Moreover, AIM2 has been linked to activation of IRF3 signaling in murine RAW264.7 cells [154]. Activation of the AIM2/ASC inflammasome seems to be dependent on the type of virus infecting the cell and possibly also the type of cell. Release of IL-1β and IL-18 is dependent on AIM2 during murine cytomegalovirus infection [129]. In contrast, AIM2 does not mediate activation of the inflammasome during HSV infection in mice [129]. Similarly, VZV activates the inflammasome independently of AIM2 in human THP-1 cells, primary lung fibroblast, and melanoma cells [118]. Overall, the data suggest that AIM2 is not a major viral DNA sensor for inflammasome activation and no evidence has linked AIM2 to virus sensing in human cells. Future studies will need to determine the AIM2-independent activation of the inflammasome in human cells and define AIM2’s role in innate signaling.

### 2.4. Other Viral Sensors and Innate Mediators

#### 2.4.1. Sensing of Viral Capsids

Viral capsids may be a general PAMP for the sensing of virus infections, and recent studies have provided evidence for innate sensing of HIV-1 and possibly adenovirus capsids. Cyclophilin A (CYPA) recognizes the HIV-1 capsids during virus assembly in human monocyte-derived DCs and subsequently CYPA mediates IRF3 signaling and activation of the DCs [131]. In addition to CYPA, TRIM5 recognizes the HIV-1 capsid (lattice) and activates NF-κB and MAPK signaling in several cells [132]. Finally, empty adenovirus capsids may trigger innate responses via an unknown mechanism when infecting the eye [163]. Future studies will need to address whether recognition of viral capsid is a broad innate sensing mechanism or limited to specific virus groups. Furthermore, it will be interesting to evaluate whether incorporation of e.g. HIV-1 capsids into vaccines would augment immune responses based on improved DC activation and antigen presentation. 

#### 2.4.2. Membrane Fusion Events

Very recent data has shown that infection with enveloped viruses may trigger virus–cell fusion events inducing innate signaling via the adaptor protein STING, including CXCL10 expression [73]. Similarly, lipid–lipid interactions have been shown to induce CXCL10 in human PBMCs and human primary macrophages [74]. The mechanism is not clear, but Holm. *et al*. exclude the involvement of DNA, RNA, and the viral capsid [73]. Also membrane penetration seems to play a role during recognition of the nonenveloped adenovirus sensed by NLRP3 [75]. 

#### 2.4.3. HMBG1

High mobility group box (HMGB) proteins are nucleotide-binding proteins localized within cells, but secreted from macrophages and other cells during infection or stimulation with inflammatory TNF-α or IL-1 [62,173]. Studies have shown that HMGBs may also play a role in both TLR and RLR recognition of pathogens [174]. HMBG1–3 act as a sensor of nucleic acids and participate in TLR3, TLR7 and TLR9 recognition of their respective nucleic acid ligands [174]. HMGBs also acts upstream of RIG-I and MDA5 promoting IFN-β after introduction of viral DNA to the cell cytoplasm, evidenced by the finding that siRNA knockdown of HMGBs impaired the IFN-β response to HSV-1 and VV genomic DNA [174]. However, the mechanism of RNA and DNA-binding to HMGBs remains to be determined. HMGB1 may also promote virus replications, since the presence of HMGB1 seems important for influenza virus and borna diseases virus replication [175,176]. In addition, HMBG1 released from DCs and NK cells induces HIV-1 replication in DCs and latently infected PBMCs, but HMBG1 inhibits the replication of HIV-1 in monocytic cells [177,178,179]. Since HMGB1 levels are increased in chronic HIV patients and associated with high viral load [180,181,182], one might speculate that exogenous HMGB1 actively modifies viral replication and viral reservoirs *in vivo*.

## 3. Innate Signaling Restricting Virus Infection

Intracellular signaling induced downstream of PRRs results in produced IFN and upregulated endogenous factor (Figure 3). Albeit the sensors that initiate antiviral responses may be located both at the cell surface, in endosomes, in the cytoplasm, or in the nucleus the initiated signaling cascades often converges at several points [2,183,184]. In the following section, virus-activated signaling pathways and the regulation of innate responses are outlined.

### 3.1. TLR Signaling

Binding of virus PAMPs to TLRs activate signaling that ultimately lead to activation of a number of the transcription factors, including IRF3, IRF7, NF-κB, and activated protein 1 (AP-1) [2,183]. TLR3 specifically signals through the adaptor protein TRIF, whereas all other TLRs signal via the adaptor protein myeloid differentiation factor 88 (MyD88). TLR4 may utilize both TRIF and MyD88. The signaling complexes associated with the adaptor molecules TRIF and MyD88 include TNF receptor-associated factor 6 (TRAF6), TRAF3, and proteins of interleukin-1 receptor-associated kinase (IRAK) family [2,183].

### 3.2. Signaling from RLRs and Cytoplasmic DNA Receptors

RNA is recognized by RIG-I and MDA5 via their C-terminal RNA binding domain. Binding of RNA results in conformational changes allowing interaction with MAVS via the RLR’s N-terminal CARDs. Interaction between MAVS and the RLRs results in formation of a protein complex, that includes TRAF6 and TRAF3, as well as stimulator of IFN genes (STING) (also known as mediator of IRF3 activation (MITA)), subsequently inducing downstream signaling [2,183]. Downstream signaling pathways lead to IRF3 and IRF7 activation via TRAF family member-associated NF-κB activator binding kinase 1 (TBK1) and IκB kinase ε (IKKε). NF-κB is activated via the IKKα/β complex and AP-1 is activated via JNK/p38/ERK MAPK pathways. A number of proteins have been reported as negative regulators of RIG-I, including the IFN-inducible protein ISG15, and the virus-inducible NF-κB regulated ubiquitin-editing protein A20 [185,186]. Present data indicate that STING mediates signaling after sensing of DNA in the cytoplasm, including recognition via the DNA receptors IFI16 and DAI, and the RNA/DNA receptor DDX41 [123,127,187,188,189].

### 3.3. Transcriptional Regulation of IFNs, Cytokines, and ISGs

Promoters of IFNs contain binding sites for members of the IFN regulatory factors (IRFs), in particular IRF3 and IRF7 [2,190]. Cytokines, including proinflammatory chemokines, are diversely regulated primarily dependent on NF-κB and AP-1 sites within their promoters [191]. AP-1 transcription factors are composed of hetero- or homodimers of Fos and Jun or ATF2 and fos proteins activated via MAPK signaling pathways [191,192]. A number of cytokines and IFN-stimulated genes (ISGs), including OAS, CCL5, and CXCL10, also rely on IRF-binding sites or IFN-sensitive response element (ISRE) sites for transcriptional regulation (Figure 3) [192,193,194].

## 4. Innate Antiviral Responses

### 4.1. Antiviral IFN responses

The IFN family of cytokines consists of three classes termed types I to III. Type I (IFN-α and IFN-β) and type III IFNs (IFN-λ1–3) mediate early antiviral responses, whereas type II IFN (IFN-γ) is an important regulator of cell activity and a classical regulator of Th1 immunity. Figure 3 illustrates the main virus-induced signaling pathways and the IFN-mediated antiviral effector functions. In the following section, both antiviral IFNs and antiviral ISG effectors will be described.

#### 4.1.1. Type I IFN

Type I IFN binding to the IFN receptor 1 (IFNR1)/IFNR2 complex activates STAT1 and STAT2 by phosphorylation via receptor-associated tyrosine kinases JAK1 and Tyk2. The phosphorylated STAT1 and STAT2 proteins dimerize and translocate to the nucleus, and activate transcription via binding to gamma activation sites (GAS) or after association with IRF9 (p48) binding to ISRE [195] leading to upregulation of hundreds of IFN-stimulated genes ISGs [196], including antiviral proteins oligoadenylate synthetase (OAS), Myxovirus resistance (Mx) genes, PKR, IFN-stimulated gene 15 (ISG15), and apolipoprotein B-mRNA editing enzyme (APOBEC) [197].

#### 4.1.2. Type III IFN

Type III IFN binds to the IL-10 receptor/IFN-λ receptor 1 (IFNRL1) complex Type III IFN (IFN-λ) display IFN type I-like biological activities [198,199] possessing direct antiviral effects similar to type I IFN at high concentration, and IFI type III possesses superior effects to type I IFN in a mucosal HSV model [200,201,202]. IFN-λ is secreted during virus infections, including influenza virus, HSV, and measles virus infection [203,204,205,206], and efficiently restricting replication of human pathogen viruses, including HIV-1, HBV, HCV, HSV-1, and HSV-2 [202,204,207,208]. It should be noted that the IFN-λ receptor, in contrast to the type I IFN receptor, is largely limited to epithelial cells and keratinocytes, thus suggesting IFN-λ’s antiviral role is restricted to sites of virus entry, such as the genital mucosal, the skin and lung epithelia [209,210].

#### 4.1.3. OAS Proteins

OAS proteins 1–3 are characterized by their ability to synthesize 2’-5’ phosphodiester bonds polymerizing ATP into 2’-5’ adenosine oligomers, which subsequently activates RNAseL that degrades ssRNA [211,212,213]. Expression of OAS is induced by type I and type III IFNs, as well as virus infections [194,214]. OASs are activated by EBV-encoded RNA (EBER-1) from EBV, adenoviral virus-associated type I (VAI) RNA from adenoviruses, and TAR RNA from HIV-1 [215,216,217]. Human genetic studies have revealed that OASs are particularly important for controlling flavivirus infections, such as WNV and HCV [218,219,220,221,222]. Further suggesting a role for OAS in viral immune defense, a SNP in OAS has been linked to affecting the immune response generated from a live-attenuated rubella vaccine [223]. Alternative functions have also been ascribed to both OAS and RNAseL. OAS released from cells has recently been shown to possess RNAseL-independent antiviral activity [224] and RNAseL has been identified as a modulator of IFN induction via cleavege of self-RNAs, which subsequently induce IFN expression via MDA5 and RIG-I [225]. 

#### 4.1.4. ISG15

The IFN-inducible, ubiquitin-like protein ISG15 mediates resistance to influenza A and B virus and HSV infection in mice and inhibition of retrovirus release from cells via interference with the protein budding complex [226,227,228]. ISG15 interferes with influenza virus at several places by inhibiting viral NS1 protein thus relieving the NS1-mediated inhibition of IFN induction via RLRs [229]. In addition, ISG15 mediates antiviral responses against dengue virus, WNV, and HIV-1 [230,231]. Data suggest that ISG15 inhibits late stages of HIV infection targeting assembly and release [231]. ISG15 may possibly support antiviral responses through the stabilization of the transcription factor IRF3 [232] important for IFN, ISG, and cytokine responses. Although ISG15 is an important antiviral protein with antiviral effect against both DNA and RNA viruses, others are not affected. For example, VSV or the arenavirus lymphocytic choriomeningitis virus (LMCV) are not inhibited by ISG15 [233]. 

#### 4.1.5. Mx Proteins

The IFN-inducible Mx family consists of Mx1 and Mx2 in mice and MxA and MxB in humans. Of the human Mx proteins, only MxA has been shown to possess antiviral activity. The antiviral activity is rather broad targeting diverse types of RNA viruses, including coxsackie virus, influenza virus, and HBV [234,235,236], probably by targeting viral nucleocapsid structures to sequester them for degradation [237,238]. In humans, SNPs in the MxA promoter is correlated with the IFN response against HCV, suggesting MxA to facilitate a major part of the anti-HCV response during IFN therapy [239,240]. Finally, a single report has linked MxA with antiviral effect against HBV [235]. The authors surprisingly found that the MxA protein did not interfere with HBV nucleocapsid assembly, but rather inhibited export of viral mRNA from the nucleus to the cytoplasm, thus suggesting a new antiviral effect of MxA.

#### 4.1.6. Viperin

Viperin (Cig5) is an IFN-inducible protein that restricts a broad range of DNA and RNA viruses, including human CMV, influenza virus, HCV, and WNV [241,242,243,244,245]. However, viperin does not seem to be a major restriction factor for HIV-1 infection [246]. Viperin is thought to inhibit virus release by budding though modification of the lipid environment within the cell or at the cell surface [242,243]. In addition to direct antiviral effects, viperin augments TLR7 and TLR9-signaling in pDCs resulting in increased IFN production [247]. Thus, viperin both directly restricts virus infections and augments antiviral IFN secretion.

#### 4.1.7. IFIT1 and IFIT2

The IFN-inducible IFIT1 (ISG56) proteins restrict virus with 5’triphosphated RNAs, such as VSV, providing both *in vitro* and *in vivo* protection [133]. Very recently, Festerl *et al.* found that IFIT2 (ISG54), but not IFIT1, protects mice from lethal neuropathology during VSV infection [248]. The finding is in contrast to results from Pichlmaier *et al.* showing a protective effect of IFIT1 against VSV infection [133]. However, IFIT1 and 2 do not protect against all neurotropic viruses, since the neurotropic RNA virus EMCV was not affected by the lack of IFIT proteins, despite being highly sensitive to IFN [248]. In conclusion, IFIT1 may be a limiting factor for some neurotrophic viruses. 

#### 4.1.8. APOBEC3

APOBEC3 proteins are capable of editing nucleic acids in ssDNAs of viral, mitochondrial, or nuclear origin [249]. The IFN-inducible APOBEC3G inhibits HIV-1 and other retroviruses through the introduction G to A hypermutations in retroviral DNA accumulating during reverse transcription [250]. APOBEC proteins may also inhibit HIV-1 via impeding reverse transcription in target cells [251,252]. The antiviral effect against HIV-1 has been shown in numerous systems, including Hela cells, pDCs, and brain endothelial cells [250,253,254]. In addition, APOBEC3C-mediated hyperediting of viral DNA has been observed for HSV-1 and EBV [255]. Moreover, APOBEC proteins are antiviral against human papilloma virus (HPV) and HBV [256,257,258]. APOBEC3G has also been shown to inhibit a number of RNA viruses, such as measles virus, mumps virus, and RSV [259] probably through interactions with viral RNAs. Collectively, the present data suggest that APOBEC proteins are broad regulators of DNA viruses and reverse transcripts from HIV-1 and other retroviruses, as well as interfering with some RNA viruses.

#### 4.1.9. SAMHD1

SAM domain and HD domain-containing protein 1 (SAMHD1) is an endogenous protein inhibiting HIV-1. SAMHD1 has recently been identified as a HIV-1 restriction factor in cells of myeloid origin [260,261]. Activated SAMHD1 restricts HIV-1 replication by depleting the intracellular pool of deoxynucleotide triphosphates through degradation of dNTPs into the composite deoxynucleoside and inorganic triphosphate [262,263].

#### 4.1.10. TRIM5α

Tripartite motif 5α (TRIM5α) is an endogenous protein known for its inhibition of retroviruses. TRIM5α is a host factor inhibiting HIV-1 at an early time point after virus entry but before reverse transcription steps, possibly *via* accelerated uncoating of the virus [264,265]. A clinical role of TRIM5α has been suggested based on the findings that polymorphisms in TRIM5α are associated with decreased susceptibility towards some strains of HIV-1 [266,267,268]. As described earlier TRIM5α recognizes HIV-1 capsids and mediates signaling via TAK1 to NF-κB and AP-1 regulating innate responses [132]. In summary, TRIM5α is an antiviral protein regulating HIV-1 infection both directly as well as indirectly via innate activation.

#### 4.1.11. Tetherin

Tetherin (also known as BST-2) is a virus- and IFN-induced glycosylated protein mainly located at the surface plasma membrane and associated with lipid rafts at the cell surface [269,270,271]. Tetherin restricts a large number of enveloped viruses, including HIV-1, HCV, KSHV, ebola virus, influenza A virus, and VSV [272,273,274,275,276,277,278,279,280]. Tetherin inhibits virus from budding from the cells and inhibits HIV-1 cell–cell spread [274,281]. However, tetherin may also promote virus infection by certain viruses, evidenced by the finding that siRNA knockdown of tetherin results in reduced CMV infection [279]. 

#### 4.1.12. TRIM22

TRIM22 (also known as Staf50) is an IFN-inducible protein restricting HIV in monocyte-derived macrophages and various cell lines [282,283,284,285]. TRIM22 inhibits HIV-1 particle production, trafficking within the host cells, or restricts HIV transcription by LTR repression [282,283,284,285]. Evidence suggests that TRIM22 also restricts other virus types, such as inhibition of HBV and EMCV gene expression and replication [286,287]. Collectively, TRIM22 is a key inhibitor of HIV-1 infection after IFN treatment, but TRIM22 may also inhibit other viruses, including the enveloped DNA virus HBV and the nonenveloped picornavirus EMCV.

#### 4.1.13. ADAR1 and ADAR2

The IFN-inducible adenosine deaminase acting on RNA 1 (ADAR1) and ADAR2 catalyzes the C-6 deamination of adenosine (A) to inosine (I) in double-stranded RNA substrates leading to destabilized RNA structures due to the mismatch of base pairs. The mismatch in base pairs may result in changes in genetic coding during viral replication, because I pairs with G and C, instead of A and U [288]. The function of ADARs is ambiguous. In some settings ADARs are inhibitory to virus infections whereas in other settings the presence of ADAR may promote virus infection. In several cell lines, ADAR1 induces mutations in HIV-1 RNA and reduces virus infectivity [289]. ADAR1 may be important for inhibition of HCV infection during IFN treatments, since IFN-α-upregulated ADAR1 limits the accumulation of self-replicating HCV RNAs in a replicon system [290]. Hepatitis delta virus (HDV) RNA is also edited by ADAR1, but not ADAR2 [291,292,293]. However, the consequence for HDV infection, which needs HBV as its helper, is not yet clear. Data suggest that ADAR1 and ADAR2 may enhance replication of a number of RNA viruses. ADAR1 and ADAR2 have both been shown to increase HIV-1 replication mainly by interaction with PKR function [294,295,296]. Similarly, ADAR1 has been shown to promote VSV infection through inhibition of PKR [297,298]. Moreover, ADAR1 inhibits measles virus-induced IFN-β and apoptosis via interference with PKR function [299,300]. Thus, ADAR1 evidently functions as a major suppressor of measles virus-induced antiviral responses and virus-induced apoptosis. Collectively, ADARs may both promote and inhibit viral replication and ADARs have an antagonistic relationship with PKR.

## 5. Therapeutic Implications of Innate Stimulation

TLR3, TLR7/8, and TLR9 have all been tested for direct antiviral properties. In addition, stimulation of other innate sensors, such as NLRs and intracellular RNA and DNA receptors, has shown promising results. It should be emphasized that many of the results described in the following section were generated using *in vitro* system and animal models. Therefore some caution should be taken when extrapolate results gained. Nevertheless, the results do provide an important foundation for future in clinical studies addressing innate activation, antipathogen responses and immune-modulation by PAMPs and by specific drugs presently used. The current knowledge on therapeutic use of PRR stimulation will be presented in the following section.

### 5.1. TLR and NLR Agonists

#### 5.1.1. TLR2 Agonists

The bacterial-derived TLR2/6 agonist FSL-1 confers *in vivo* resistance to genital infection with HSV-2 when FSL-1 is applied in the vagina [301]. The authors also found that FSL-1 attenuates HSV-2 replication in human vaginal epithelial cell cultures, suggesting that the TLR2 agonist could be used for antiviral treatment [301]. However, other studies have shown that the TLR2 agonist peptidoglycan does not enhance resistance to vaginal HSV infection [302]. As described earlier, TLR2 responses in human and murine system do not always correlate making extrapolation of results to human settings very difficult. In addition, TLR2 signaling has been shown to activate T cells to be more susceptible to productive infection with HIV-1 [303] and TLR2 agonists may increase the susceptibility to HIV transmission to T cells by DCs, a target for HIV-1 infection in genital tissues [304,305]. Thus, the application of TLR2 agonist in humans to restrict sexually transmitted diseases like HSV would possibly increase the risk of HIV infection. 

#### 5.1.2. TLR3 Agonists

TLR3 agonists have proven efficient against a number of viruses primarily via induced IFN and ISGs. Moreover TLR3 may both increases antigen uptake and cross-presentation from DCs [306]. TLR3 stimulation may, however, also reduce antigen uptake and cross presentation [307]. Engagement of TLR3 induces an antiviral state in human microglial cells and astrocytes inhibiting HIV-1 infection [308,309]. Similarly, TLR3 stimulation inhibits HIV in *ex vivo* lymphoid tissues cultures [310]. In the astrocytes, the antiviral effect of TLR3 was found to be via produced IFN-β and subsequent expression of viperin evidenced by the finding that antibodies against IFN or specific siRNA knockdown of viperin abrogated the observed inhibition of HIV-1 replication [309]. Similar findings have been gained in human genital epithelial cells infected with HSV-2 [311,312,313] and targeted stimulation of TLR3 protects mice from genital HSV-2 infection in a mouse model [311,314]. Vaginally administered TLR3 agonists induce a range of cytokines in the vagina, including type I IFN, IL-1α, IL-1β, IL-6, CCL3, and CCL5, but the antiviral effects has been directly linked to IFN-β production [302,311]. TLR3 stimulation may also provide protection against CNS infection with a neurovirulent strain of HSV, since intranasal or interperitoneal pretreatment with a TLR3 agonist reduces virus load and survival of infected mice [315]. Several compounds may improve TLR3 responses, including mixture of dsRNA with the antimicrobial peptide LL37, several cationic and cell permeable peptides, or dsRNA-binding proteins [316,317]. Finally, it is worth noting that choice of vector for delivery of either an antigen or the specific TLR3 agonist could be important for design of gene therapy, vaccines, or prophylactic treatments. One example is that lentiviral vectors trigger TLR3 and TLR7 in mice resulting in improved CD8+ T-cell antigen-specific responses [26]. TLR3 triggering via dsRNA directly activates CD4+ and CD8+ effector and memory cells [318]. Thus, TLR3 agonists could possibly be included in drug combinations for activation of HIV reservoirs during eradications studies. However, such studies should be carefully monitored for the possible activation latent virus infections, such as resting herpes virus infections and endogenous retroviruses. As will be mentioned later, TLR3 synergistically activates DCs and induces augmented CTL responses in presence of TLR7/8 ligands [319]. Mechanistically, the effect may partly rely on high IL-12 production from DCs after stimulation of TLR3 and TLR7/8 [320].

#### 5.1.3. TLR4 Agonists

TLR4 agonists are efficient inducers of Th1 responses and thus may be utilized as adjuvants for virus vaccines and treatment of allergic reactions [321]. In line with the data gained for TLR3 agonists, TLR4 synergistically acts with TLR7/8 agonists, inducing a broad range of cytokines, including IL-12, resulting in DC activation [320]. In DCs, TLR4 agonists may provide increased antigen uptake and a transient increase in cross presentation, but antigen uptake and cross-presentation may also be inhibited by TLR4 stimulation [307,322,323]. It should be noted that some TLR4 agonists seem more efficient and safe than others. The TLR4 agonist monophosphoryl lipid A (MPL) derived from *Salmonella minnesota* has successfully been added to several virus vaccines, including HBV and HPV vaccines [321]. In a murine model, pretreatment with *Escherichia coli*-derived LPS was not found protective against vaginal HSV-2 infection [302]. In contrast, another study has shown a protective effect of the *Escherichia coli*-derived TLR4 agonist Fim-H [324]. The most likely explanation for the divergent results is the IFN-β inducing capacity of Fim-H, but not LPS, in the used experimental settings [302,324].

TLR4 stimulation may have negative influence on HIV-1 infection and transmission. Engagement of TLR4 reduces HIV-1 transmission from DCs to CD4+ T cells via secreted type I IFN [305]. Similarly, LPS stimulation of macrophages and microglia cells may inhibit HIV replication [308,325]. The mechanism may be via induced IFN, since the TLR4 stimulation of the microglial cells results in IRF3 activation [308]. However, *in vitro* studies using reporter cell lines have shown that LPS may also activate HIV replication via activation of NF-κB that subsequently binds to HIV LTR promoter regions [326,327]. *Ex vivo* studies in human lymphoid tissues show that replication of macrophage trophic HIV is enhanced by the presence of LPS derived from *E. coli* [310]. The results are in line with the theory that leakage of bacteria from the gut in HIV patients induces a systemic inflammation via TLR4 activation escalating HIV-1 pathogenesis [328]. Nevertheless, TLR4 agonists do have great potential as vaccine adjuvants and as direct treatments bearing in mind the possible negative consequences of TLR4 activation. Moreover, TLR4 agonist should be carefully chosen after preevaluating the use and patient group.

#### 5.1.4. TLR7/8 Agonists

TLR7/8 agonists trigger a broad innate response, including secretion of IFN-α, TNF-α, IL-12, and IFN-γ [329,330]. TLR7/8 agonists are activators of DCs, enhancing DC survival, DC trafficking to the draining lymph nodes after vaccination, and enabling DC cross-presentation of extracellular material to CD8+ T cells [331,332,333]. Moreover, TLR7/8 stimulation triggers NK activation and IFN-γ secretion [334]. Tailored innate responses could be generated via choice of RNA structures and through combination of RNAs with liposomes for delivery to the cytoplasm [335]. Delivery of PAMPs to the cytoplasm is also discussed later. Agonists for TLR7/8 have been used for experimental treatments of HSV and HPV. In a randomized, controlled trail 0.01% resiquimod (R848) decreased genital HSV-2 shedding [336]. However, resiquimod does not efficiently inhibit acquisition of HSV-2 infection, since intravaginally administered resiquimod does not protect against genital infection in mice [337]. In contrast to the HSV-2 studies, TLR7/8 agonists administered intranasally protect against respiratory influenza virus infection in rats [338]. Finally, the TLR7 agonist loxoribine has been shown to inhibit HIV replication in ex vivo lymphoid tissues cultures [310]. Currently, a 5% imiquimod cream targeting TLR7 is approved for treatment of HPV-induced genital warts, whereas treatment of genital HSV infection with a resiquimod cream targeting TLR7/8 has been discontinued [321].

TLR7/8 agonists have successfully been included in some vaccine formulations. Experiments emulsifying or conjugating a TLR7/8 ligand to HIV-gag resulted in improved Th1 and CD8+ T cell responses [339,340]. Similarly, codelivery of TLR7/8 agonists with a Norwark virus-like particle vaccine resulted in improved systemic and mucosal immune responses [341]. Coadministration of TLR7/8 agonists with HBsAg improves humoral and cellular responses [342]. In addition, TLR7/8 activation of DCs may improve generation of virus-specific T cell responses in hosts with latent infections, such as HIV-1- and CMV-infected individuals [331]. The vehicle for vaccine antigen delivery or delivery of short interfering RNAs (siRNAs) may also trigger TLR7 and augment immune responses. One example is lentiviral vectors triggering TLR7 and activating DCs important for efficient CD8+ T-cell responses [26]. Several attempts have been made to target viral genes to restrict infections using siRNA approaches [343]. In that regard, it is interesting that some siRNAs may activate TLR7 and promote antipathogen or antitumor responses via produced IFN [344]. Bifunctional siRNAs, triggering both innate responses via TLR7 and targeting viral genes may therefore have a better therapeutic index than highly efficient gene-targeting siRNAs with no TLR7 stimulatory capability. Future studies will have to delineate whether stimulation of the immune system via TLR7 can be incorporated into successful vaccines vectors and siRNAs targeting viral genes.

It should also be noted that *in vivo* TLR crosstalk challenge the design of immune-modulators. One example is TLR7/8 triggered by HIV-1-encoded ssRNAs augments TLR4 and TLR2 responses [345,346]. Moreover, TLR ligands may synergistically activate innate responses. dsRNA and resiquimod, for example, synergistically activate DCs and increase the CTL responses generated after vaccination with DCs pulsed with peptides [319]. Thus, the stimulation of one TLR receptor may affect the immune response generated through other receptors, combinations of TLR agonists may provide synergistic effects, and targeted stimulation of one or several TLRs may be utilized to enhance antigen-specific responses or antiviral immune-stimulation.

#### 5.1.5. TLR9 Agonists

TLR agonists are potent inducers of Th1-orientated immune responses and TLR9 ligands may promote antigen uptake and cross presentation [306,347]. Studies have shown that choice of CpG oligodeoxynucleotides (ODNs) affects the innate response triggered. Accordingly, CpG ODNs have been classified based on their response. Class A CpG ODNs are more potent inducer of IFN-α, whereas class B CpG ODNs are stronger inducer of proinflammatory IL-8 and enhanced expression of activation molecules CD80 and CD86, as well as antigen-presenting MHC II [347,348]. Importantly, a report suggests that CpG B ODNs may also inhibit TLR-dependent and TLR-independent IFN responses via an unknown mechanism [26]. CpG DNA has been proposed as therapy for several infections, including HSV-2 and HIV-1 [337,349,350,351]. Vaginally administrated CpG DNA provides protection against genital HSV-2 infection in a mouse model [337,352]. TLR9 agonists are included in vaccines to provide an adjuvant effect. A positive adjuvant effect of CpG DNA has been identified for several vaccines, including hepatitis B surface antigen (HBsAg), HSV-2 antigens, and influenza virus antigens [321,342,350].

Although stimulation of PRRs may reduce virus burden via enhanced production of IFNs, cytokines and intrinsic antiviral factors, triggering of cell PRRs may also enhance virus replication. For example, triggering of TLR4 and TLR9 or intracellular DNA receptor DAI may activate NF-κB and enhance HIV replication [152,353]. Moreover, CpG DNA may activate CD4+ cells [318], and thus enhance the number of target cells for HIV. However, the data is not unambiguous, since TLR9 stimulation may also inhibit HIV replication in lymphoid tissue [310]. Data from our group show that time of administration of a TLR9 adjuvant during vaccination may strongly affect the generated immune response [354]. Therefore, a timely and controlled stimulation of innate receptors is required to elicit effective antipathogen responses and avoid detrimental immune reactions. TLR9-mediated activation of HIV and activation of T cells may, however, also be utilized to activate HIV from resting T cells in eradication studies. Moreover, TLR9 stimulation may possibly increase antigen-specific T cell responses in HIV-1- and CMV-infected individuals [331]. TLR9 agonist may be incorporated into the vector used, since lentiviruses pseudotyped with VSV-G protein containing tubulovesicular structures derived from the host cell may trigger an antiviral response via TLR9 [355]. In addition, TLR9 mediates recognition of adenoviral vector DNA in pDCs resulting in innate immune responses, including type I IFN production [356]. Collectively, more research is needed to understand the functional interplay between innate responses, virus replication, and interactions with adaptive response, but TLR agonists and combinations thereof are promising candidates for novel therapies.

#### 5.1.6. NLR Agonists

As previously described, NOD2 and NLRP3 recognize RNA viruses, including influenza A virus and RSV [111,112]. Stimulation of NOD2 results in an efficient antiviral response against influenza A virus in mice [357]. Postinfection intravenous treatment with bacterial-derived muramyl dipeptide (MDP) targeting NOD2 protects mice from influenza A virus infection via induced IFN-β and CCL2 and recruitment of inflammatory monocytes to the lungs [357]. In addition, NOD2 and TLR9 agonists administered together with inactivated RSV improve the mucosal and systemic immunity when compared to inactivated RSV alone [358]. Overall, the data suggest NOD2 stimulation may positively be utilized alone or in combination with other PRR agonists to generate or improve antiviral responses. 

### 5.2. Targeting Cytoplasmic DNA Receptors

As described previously, activation of DNA and RNA sensors in the cell’s cytoplasm result in IFN responses, as well as secretion of proinflammatory cytokines important for efficient control of virus infections. Targeting one or several DNA or RNA receptors may therefore be an attainable way to reduce virus infection and to elicit efficient vaccine responses. One example hereof is that targeted delivery of dsRNA and DNA-using liposomes results in strong immune activation, including DC maturation, and improved T cells responses in mice [359]. In mouse and guinea pig models, a potent adjuvant effect has been observed using DNA-liposome complexes together with a HSV vaccine [360,361]. Moreover, adenoviral vector DNA is sensed by a cytoplasmic DNA sensor in non-pDC cells, resulting in innate responses, including type I IFN expression [356]. Finally, modified VV Ankara, often used as a vaccine vector, may activate MDA5 and the NLRP3 inflammasome, resulting in IFN responses and IL-1β processing, respectively [362].

Targeting of the DNA receptor DAI has been shown to promote an adjuvant effect to DNA vaccines [363]. However, other studies suggest no role of DAI for recognition of DNA vaccines [364].

Very recent data suggest that lipid–lipid interactions may trigger innate responses, including CXCL10 expression [73,74]. Therefore, DNA-liposome adjuvant effects may derive from two signals: one via lipid interaction with cell surfaces, and one signal from the recognized DNA in the cytoplasm. In addition to lipid administration of DNA, nanoparticles may be used to target DNA to cells possibly targeting DNA selectively to TLRs or cytoplasmic DNA receptors. Initial studies have shown that DNA/polyethylenimine nanoparticles generate robust proinflammatory responses via both TLR9-dependent and TLR9-independent mechanisms [365]. Future research should delineate how DNA receptors may selectively be activated and which responses they induce *in vivo*.

## 6. Immunomodulatory Effect of Antimicrobial Drugs

Currently used antivirus treatments may modulate innate and adaptive immune responses. The choice and timing of drug regiments could significantly influence the direct antiviral response raised against invading virus. General antimicrobial chemotherapy may for instance negatively influence general antiviral responses via change of the normal flora. Furthermore, the outcome of secondary infections in, for instance, HIV patients could be affected by choice of treatments. Several drug regiments affect cytokine networks and thereby mediate changes in generated immune responses. The following section will briefly discuss and exemplify how some antimicrobial drugs affect antiviral immune responses.

### 6.1. Antiviral Treatment/HAART

#### 6.1.1. Proteases Inhibitors

Protease inhibitors used in HIV and HCV therapy interferes with NF-κB activation and thus inhibits production of proinflammatory cytokines during stimulation via TLR2 and TLR4 [366]. The proteases inhibitor nelfinavir may also inhibit MAPK signaling and thus AP-1 activation [367]. Protease inhibitors may, however, also enhance expression of certain proinflammatory cytokines during virus infection, evidenced by the finding that IL-8 mRNA accumulation is synergistically increased in oral keratinocytes by synthetic dsRNA and presence of the HIV-1 protease inhibitor lopinavir [368]. The mechanism of action for NF-κB inhibition may be via interaction with the proteasome [369], important for degradation of inhibitor κB restricting NF-κB translocation to the nucleus and thus NF-κB activation. Overall, the majority of data suggest that protease inhibitors may dampen inflammation and thus could provide some additional benefits dampening detrimental inflammation during HIV infection or HCV infection.

#### 6.1.2. RT Inhibitors

The HIV reverse-transcriptase (RT) inhibitor azidothymidine (AZT) reportedly affects cells differently. AZT enhances NF-κB activation in a promyeloide leukemia cell line, the monocyte-like U937 cell line, and the T lymphoblast cell line MOLT [370]. AZT also enhance IL-8 and CCL3 secretion from U937 cells after TLR-stimulation or infection with bacteria [371]. In contrast, AZT inhibits NF-κB activation and induces EBV expression in Burkitt’s lymphoma B cells [372]. Another RT inhibitor abacavir induces early changes in cells, including redistribution of heat-shock protein 70 [373]. The RT inhibitor tenofovir, used in HIV and HCV treatments also affects innate responses *in vitro* [371,374]. The oral prodrug of tenofovir, tenofovir disproxyl fumerate (TDF) inhibits TLR-mediated and CMV- and bacteria-mediated activation production of proinflammatory IL-8 and CCL3 in human monocytes and PBMC cultures [371]. Importantly, TDF also enhances the ability to produce IL-12 after TLR or bacterial challenge in human and macaque PBMCs, thus possibly promoting generation of CTL responses important for virus control [371,374]. Tenofovir has been shown to induce accumulation of IL-1β, TNF-α, CCL3, CCL5 and IL-10, but not IL-12 and IFN-γ, in murine peritoneal cell culture [375] and CCL3 and CCL5 in human PBMCs [376]. Tenofovir has drawn attention, since the drug formulated in a microbicide for vaginal application was the very first to reduce HIV-1 acquisition in a clinical setting [377] and surprisingly also reduced HSV-2 acquisition [377,378]. Recently, tenofovir and the prodrug TDF were found to be directly antiviral against HSV-2 [379,380,381], which could explain the success of tenofovir in the clinical microbicide trail. In conclusion, antiviral drugs may both inhibit and activate secretion of cytokines and interfere with cell signaling.

#### 6.1.3. Antibiotics

#### 6.1.4. Macrolides—Activators and Inhibitors of Innate Responses

Macrolides are used to treat infection with bacterial infections, such as respiratory infection with *Streptococcus pneumoniae* and *Haemophilus influenzae*. The macrolide clarithromycin has been shown to suppress IL-8 production from human monocyte-like THP-1 cells [382]. Similarly, erythromycin inhibits IL-6 and IL-8 secretion from human bronchial epithelial cells during *Haemophilus influenza* infection [383]. Similarly, erythromycin inhibits TNF-α and IL-8 secretion from *Streptococcus pneumonia*-treated whole blood and TNF-α from *pseudomonas aeruginos*-treated whole blood [384,384]. The presence of azithromycin is also suppressive measured by decreased expression of IL-1β and TNF-α after TLR2-stimulation of human corneal epithelial cells [385]. Similarly, azithromycin treatment of primary bronchial epithelial cells attenuates the cells ability to produce IL-8 and granulocyte macrophage colony-stimulating factor (GM-CSF) after LPS stimulation [386]. Since elevated expression of several cytokines, including IL-8, IL-6, GM-CSF, is seen during pulmonary infections the macrolides could provide additional protection from immune-pathogenesis via their anti-inflammatory properties.

Several macrolides may also shift the balance of Th1 and Th2 cytokines, evidenced by the finding that clarithomycin, midecamycin acetate, and josamycin potently inhibit TNF-α and IL-2 release from mitogen-stimulated T cells, whereas IFN-α, IL-4, IL-5, IL-6, and IL-10 are only slightly suppressed [387]. Macrolides may also suppress IL-12 and increase IL-10, since azithromycin treatment results in increased amount of IL-10 and decreased amount of IL-12 and TNF-α after LPS and IFN-γ stimulation of murine macrophage-like J774 cells [388]. In conclusion, the data suggest that macrolides affects cells differently dependent on concentration, cell type, and the specific macrolide used.

Antimicrobial treatment may also activate innate response. The macrolides nystatin, and natamycin and the antifungal drug amphotericin B may directly activate the NLRP3 inflammasome in murine bone marrow-derived DCs via intracellular release of potassium leading to IL-1β secretion and increased inflammation [389]. Similarly, IL-1β secretion from human THP-1 cells has been observed after stimulation with nystatin and amphotericin [390,391]. In addition to NLRP3 activation, nystatin and amphotericin B mediate production of proinflammatory cytokines IL-8, IL-6, and TNF-α viadirect recognition via TLR2 in human monocytic THP-1 cells and HEK293 cells [390,391]. Thus, some antibacterial and antifungal drugs are efficient activators of innate immunity, but may also inhibit innate responses. It remains to be determined whether the difference in responses may be due to timing, concentration, and cells used.

#### 6.1.5. Other Antibiotics and Effects on Virus Diseases

Metronidazole (MTZ) is used to treat anaerobic bacteria and protozoa also possess immune-stimulatory effects. MZT has been shown to inhibit production of IL-1β, IL-6, IL-8, IL-12, and TNF-α in human oral cavity cells treated with either LPS or *Porphyromonas gingivalis* a gram-negative bacteria associated with periodontal diseases characterized by a detrimental inflammatory process [392]. Clindamycin is a lincosamide antibiotic for treatment of anaerobic bacteria and some protozoan diseases. Clindamycin inhibits expression of TNF-α, IL-6, and IL-1β in murine peritoneal macrophages after LPS stimulation [393]. The findings translate to *in vivo* studies showing that the presence of clindamycin renders mice less susceptible to endotoxic shock [394]. Telithromycin is a semisynthetic, ketolide for treatment of bacterial, respiratory infections. Telithromycin also inhibits the secretion of the inflammatory mediators IL-1α and TNF-α, but not IL-1β, IL-6, and IL-10, after LPS stimulation of human peripheral blood monocytes [395]. Polymyxin B is an antibiotic primarily used for treatment of gram-negative infections. Polymyxin B induces hepatocyte growth factor (HGF) production in human dermal fibroblast via a MAPK-dependent mechanism [396]. The finding is interesting because HGF is a growth factor for keratinocytes and polymyxin is commonly used as a topical antibiotic for wound care. The induction of a HFG may possibly promote tissue repair and simultaneously reduce the risk of infection.

A direct effect of antibiotics on viral diseases has been observed to be mediated through disruption of the natural microbiota in the body. Studies in mice have revealed that antibiotic-mediated disruption of the microbiota severely impairs antiviral response against influenza A virus infections [397]. The aminoglycoside neomycin, but not ampicillin, vancomycin, or metronidazole, impaired the antiviral response against the virus. The mechanism is thought to be decreased expression of pro-IL-1β and pro-IL-18 due to decreased levels of commensal bacteria and thus impaired IL-1β and IL-18 secretion via the NLRP3 inflammasome ultimately leading to reduced DC activity and attenuated T cells priming and CTL response [397]. Interestingly, the authors found that rectal or nasal TLR stimulation could restore immune response to influenza virus in the antibiotic-treated mice emphasizing that commensal bacteria provide important signals for PRRs.

The inflammatory response generated during bacterial and fungal infections is both important for a strong antipathogen response the inflammatory response may also be harmful. Timed and targeted use of antibiotics with high or low or non-immuno-modulatory effect with could be beneficial in some patients during infections. However, use of antibiotics with immuno-modulatory effects should be carefully monitored, and further investigations should delineate the optimal use of the individual drugs and how they work in concert. The use of antimicrobial agents with additional anti-inflammatory effects may be beneficial for development of topical treatments, including development of microbicides against sexually transmitted infections (STIs). However, it should be noted that results gained in cell cultures and animal studies are very preliminary and not easily extrapolated into humans.

## 7. Concluding Remarks

Innate immune activation, including IFN responses, seems to be a double-edged sword positively affecting virus control, but at the same time contributing to virus pathogenesis. Detailed knowledge of the innate sensing pathways and generated cytokine and antiviral responses may provide targets for intervention. Knowledge of innate immunity is important to fill in gaps in understanding of viral pathogenesis and why some vaccines work and other do not. Improved knowledge is especially important for development of vaccines for viruses where no vaccine is currently available.

Modulation of innate responses has proven important for new efficient vaccine strategies, and traditional vaccine adjuvants have shown to modulate innate responses and subsequent adaptive responses. However, major challenges lie ahead of us in deciphering the innate sensing mechanism and figuring out how we can regulate the antiviral responses. Moreover, when developing intervention strategies, we have to take into account that viruses employ multiple evading mechanisms employed by viruses. Carefully designed drugs triggering innate responses may indeed give rise to new therapeutics, such as prophylaxis, topical treatments, inhalation of drugs, or microbicides. Moreover, the use of drugs in carefully chosen combinations may provide improved treatments. The combination of drugs or PAMPS in nanoparticles or lipid combinations provide new ways of triggering innate responses together with targeted delivery of the drug or PAMP.

The present data suggest that drugs and innate vaccine adjuvant should be carefully dosed and timed during vaccination programs. Moreover, the use of known antimicrobial treatments should be carefully chosen. Many central questions remain to be answered to integrate the knowledge on innate virus–host interactions into development of new vaccination strategies and novel treatment regimens beneficial to patients.

Finally, data has emerged that current antiviral and antibacterial therapies may affect control of innate and adaptive immune-response. It should be noted that most data has been gained in cell cultures and mouse studies and thus the effects might not be of clinical significance in humans. However, in order to improve treatments, the present data do warrant need for further studies in the field of immune-modulatory effects in humans.

## References

[1] Ishii K.J., Koyama S., Nakagawa A., Coban C., Akira S. (2008). Host innate immune receptors and beyond: Making sense of microbial infections. Cell Host Microbe..

[2] Takeuchi O., Akira S. (2010). Pattern recognition receptors and inflammation. Cell.

[3] Kim H.M., Park B.S., Kim J.I., Kim S.E., Lee J., Oh S.C., Enkhbayar P., Matsushima N., Lee H., Yoo O.J. (2007). Crystal structure of the TLR4-MD-2 complex with bound endotoxin antagonist Eritoran. Cell.

[4] Kurt-Jones E.A., Chan M., Zhou S. (2004). Herpes simplex virus 1 interaction with Toll-like receptor 2 contributes to lethal encephalitis. Proc. Natl. Acad. Sci. U.S.A..

[5] Leoni V., Gianni T., Salvioli S., Campadelli-Fiume G. (2012). Herpes Simplex Virus Glycoproteins gH/gL and gB Bind Toll-Like Receptor 2, and Soluble gH/gL Is Sufficient To Activate NF-kappaB. J. Virol..

[6] Boehme K.W., Guerrero M., Compton T. (2006). Human cytomegalovirus envelope glycoproteins B and H are necessary for TLR2 activation in permissive cells. J. Immunol..

[7] Compton T., Kurt-Jones E.A., Boehme K.W. (2003). Human cytomegalovirus activates inflammatory cytokine responses via CD14 and Toll-like receptor 2. J. Virol..

[8] Gaudreault E., Fiola S., Olivier M., Gosselin J. (2007). Epstein-Barr virus induces MCP-1 secretion by human monocytes via TLR2. J. Virol..

[9] Ariza M.E., Glaser R., Kaumaya P.T., Jones C., Williams M.V. (2009). The EBV-encoded dUTPase activates NF-kappa B through the TLR2 and MyD88-dependent signaling pathway. J. Immunol..

[10] Wang J.P., Kurt-Jones E.A., Shin O.S., Manchak M.D., Levin M.J., Finberg R.W. (2005). Varicella-zoster virus activates inflammatory cytokines in human monocytes and macrophages via Toll-like receptor 2. J. Virol..

[11] Bieback K., Lien E., Klagge I.M. (2002). Hemagglutinin protein of wild-type measles virus activates toll-like receptor 2 signaling. J. Virol..

[12] Dolganiuc A., Oak S., Kodys K. (2004). Hepatitis C core and nonstructural 3 proteins trigger toll-like receptor 2-mediated pathways and inflammatory activation. Gastroenterology.

[13] Zhu J., Martinez J., Huang X., Yang Y. (2007). Innate immunity against vaccinia virus is mediated by TLR2 and requires TLR-independent production of IFN-beta. Blood.

[14] Martinez J., Huang X., Yang Y. (2010). Direct TLR2 signaling is critical for NK cell activation and function in response to vaccinia viral infection. PLoS Pathog.

[15] Kurt-Jones E.A., Popova L., Kwinn L. (2000). Pattern recognition receptors TLR4 and CD14 mediate response to respiratory syncytial virus. Nat. Immunol..

[16] Triantafilou K., Triantafilou M. (2004). Coxsackievirus B4-induced cytokine production in pancreatic cells is mediated through toll-like receptor 4. J. Virol..

[17] Georgel P., Jiang Z., Kunz S. (2007). Vesicular stomatitis virus glycoprotein G activates a specific antiviral Toll-like receptor 4-dependent pathway. Virology.

[18] Zhang S.Y., Jouanguy E., Ugolini S. (2007). TLR3 deficiency in patients with herpes simplex encephalitis. Science.

[19] Iwakiri D., Zhou L., Samanta M. (2009). Epstein-Barr virus (EBV)-encoded small RNA is released from EBV-infected cells and activates signaling from Toll-like receptor 3. J. Exp. Med..

[20] Alexopoulou L., Holt A.C., Medzhitov R., Flavell R.A. (2001). Recognition of double-stranded RNA and activation of NF-kappaB by Toll- like receptor 3. Nature.

[21] Guillot L., Le Goffic R., Bloch S. (2005). Involvement of toll-like receptor 3 in the immune response of lung epithelial cells to double-stranded RNA and influenza A virus. J. Biol. Chem..

[22] Le Goffic R., Balloy V., Lagranderie M. (2006). Detrimental contribution of the Toll-like receptor (TLR)3 to influenza A virus-induced acute pneumonia. PLoS Pathog.

[23] Le Goffic R., Pothlichet J., Vitour D. (2007). Cutting Edge: Influenza A virus activates TLR3-dependent inflammatory and RIG-I-dependent antiviral responses in human lung epithelial cells. J. Immunol..

[24] Rudd B.D., Burstein E., Duckett C.S., Li X., Lukacs N.W. (2005). Differential role for TLR3 in respiratory syncytial virus-induced chemokine expression. J. Virol..

[25] Rudd B.D., Smit J.J., Flavell R.A. (2006). Deletion of TLR3 alters the pulmonary immune environment and mucus production during respiratory syncytial virus infection. J. Immunol..

[26] Breckpot K., Escors D., Arce F. (2010). HIV-1 lentiviral vector immunogenicity is mediated by Toll-like receptor 3 (TLR3) and TLR7. J. Virol..

[27] Wang Q., Miller D.J., Bowman E.R. (2011). MDA5 and TLR3 initiate pro-inflammatory signaling pathways leading to rhinovirus-induced airways inflammation and hyperresponsiveness. PLoS Pathog.

[28] Wang Q., Nagarkar D.R., Bowman E.R. (2009). Role of double-stranded RNA pattern recognition receptors in rhinovirus-induced airway epithelial cell responses. J. Immunol..

[29] Wang T., Town T., Alexopoulou L., Anderson J.F., Fikrig E., Flavell R.A. (2004). Toll-like receptor 3 mediates West Nile virus entry into the brain causing lethal encephalitis. Nat. Med..

[30] Daffis S., Samuel M.A., Suthar M.S., Gale M., Diamond M.S. (2008). Toll-like receptor 3 has a protective role against West Nile virus infection. J. Virol..

[31] Beignon A.S., McKenna K., Skoberne M. (2005). Endocytosis of HIV-1 activates plasmacytoid dendritic cells via Toll-like receptor-viral RNA interactions. J. Clin. Invest..

[32] Alter G., Suscovich T.J., Teigen N. (2007). Single-stranded RNA derived from HIV-1 serves as a potent activator of NK cells. J. Immunol..

[33] Meier A., Alter G., Frahm N. (2007). MyD88-dependent immune activation mediated by human immunodeficiency virus type 1-encoded Toll-like receptor ligands. J. Virol..

[34] Lund J.M., Alexopoulou L., Sato A. (2004). Recognition of single-stranded RNA viruses by Toll-like receptor 7. Proc. Natl. Acad. Sci. U.S.A..

[35] Melchjorsen J., Jensen S.B., Malmgaard L. (2005). Activation of innate defense against a paramyxovirus is mediated by RIG-I and TLR7 and TLR8 in a cell-type-specific manner. J. Virol..

[36] Triantafilou K., Orthopoulos G., Vakakis E. (2005). Human cardiac inflammatory responses triggered by Coxsackie B viruses are mainly Toll-like receptor (TLR) 8-dependent. Cell Microbiol..

[37] Lund J., Sato A., Akira S., Medzhitov R., Iwasaki A. (2003). Toll-like receptor 9-mediated recognition of Herpes simplex virus-2 by plasmacytoid dendritic cells. J. Exp. Med..

[38] Krug A., Luker G.D., Barchet W., Leib D.A., Akira S., Colonna M. (2004). Herpes simplex virus type 1 activates murine natural interferon-producing cells through toll-like receptor 9. Blood.

[39] Rasmussen S.B., Sorensen L.N., Malmgaard L. (2007). Type I IFN production during herpes simplex virus infection is controlled by cell-type specific viral recognition through TLR9, the MAVS pathway, and novel recognition systems. J. Virol..

[40] Sato A., Linehan M.M., Iwasaki A. (2006). Dual recognition of herpes simplex viruses by TLR2 and TLR9 in dendritic cells. Proc. Natl. Acad. Sci. U.S.A..

[41] Malmgaard L., Melchjorsen J., Bowie A.G., Mogensen S.C., Paludan S.R. (2004). Viral activation of macrophages through TLR-dependent and -independent pathways. J. Immunol..

[42] Varani S., Cederarv M., Feld S. (2007). Human cytomegalovirus differentially controls B cell and T cell responses through effects on plasmacytoid dendritic cells. J. Immunol..

[43] Yu H.R., Huang H.C., Kuo H.C. (2011). IFN-alpha production by human mononuclear cells infected with varicella-zoster virus through TLR9-dependent and -independent pathways. Cell Mol. Immunol..

[44] Lim W.H., Kireta S., Russ G.R., Coates P.T. (2007). Human plasmacytoid dendritic cells regulate immune responses to Epstein-Barr virus (EBV) infection and delay EBV-related mortality in humanized NOD-SCID mice. Blood.

[45] Fiola S., Gosselin D., Takada K., Gosselin J. (2010). TLR9 contributes to the recognition of EBV by primary monocytes and plasmacytoid dendritic cells. J. Immunol..

[46] West J.A., Gregory S.M., Sivaraman V., Su L., Damania B. (2011). Activation of plasmacytoid dendritic cells by Kaposi's sarcoma-associated herpesvirus. J. Virol..

[47] Samuelsson C., Hausmann J., Lauterbach H. (2008). Survival of lethal poxvirus infection in mice depends on TLR9, and therapeutic vaccination provides protection. J. Clin. Invest..

[48] Basner-Tschakarjan E., Gaffal E., O'Keeffe M. (2006). Adenovirus efficiently transduces plasmacytoid dendritic cells resulting in TLR9-dependent maturation and IFN-alpha production. J. Gene. Med..

[49] Appledorn D.M., Patial S., McBride A. (2008). Adenovirus vector-induced innate inflammatory mediators, MAPK signaling, as well as adaptive immune responses are dependent upon both TLR2 and TLR9 *in vivo*. J. Immunol..

[50] Awomoyi A.A., Rallabhandi P., Pollin T.I. (2007). Association of TLR4 polymorphisms with symptomatic respiratory syncytial virus infection in high-risk infants and young children. J. Immunol..

[51] Hutchens M.A., Luker K.E., Sonstein J., Nunez G., Curtis J.L., Luker G.D. (2008). Protective effect of Toll-like receptor 4 in pulmonary vaccinia infection. PLoS Pathog.

[52] Imai Y., Kuba K., Neely G.G. (2008). Identification of oxidative stress and Toll-like receptor 4 signaling as a key pathway of acute lung injury. Cell.

[53] Barbalat R., Lau L., Locksley R.M., Barton G.M. (2009). Toll-like receptor 2 on inflammatory monocytes induces type I interferon in response to viral but not bacterial ligands. Nat. Immunol..

[54] Kijpittayarit S., Eid A.J., Brown R.A., Paya C.V., Razonable R.R. (2007). Relationship between Toll-like receptor 2 polymorphism and cytomegalovirus disease after liver transplantation. Clin. Infect. Dis..

[55] Ahmad R., El B.S., Cordeiro P., Menezes J. (2008). Requirement of TLR2-mediated signaling for the induction of IL-15 gene expression in human monocytic cells by HSV-1. Blood.

[56] Mansur D.S., Kroon E.G., Nogueira M.L. (2005). Lethal encephalitis in myeloid differentiation factor 88-deficient mice infected with herpes simplex virus 1. Am. J. Pathol..

[57] Bochud P.Y., Magaret A.S., Koelle D.M., Aderem A., Wald A. (2007). Polymorphisms in TLR2 are associated with increased viral shedding and lesional rate in patients with genital herpes simplex virus Type 2 infection. J. Infect. Dis..

[58] Sorensen L.N., Reinert L.S., Malmgaard L., Bartholdy C., Thomsen A.R., Paludan S.R. (2008). TLR2 and TLR9 synergistically control herpes simplex virus infection in the brain. J. Immunol..

[59] Reske A., Pollara G., Krummenacher C., Katz D.R., Chain B.M. (2008). Glycoprotein-dependent and TLR2-independent innate immune recognition of herpes simplex virus-1 by dendritic cells. J. Immunol..

[60] Piccinini A.M., Midwood K.S. (2010). DAMPening inflammation by modulating TLR signalling. Mediators Inflamm..

[61] Wheeler D.S., Chase M.A., Senft A.P., Poynter S.E., Wong H.R., Page K. (2009). Extracellular Hsp72, an endogenous DAMP, is released by virally infected airway epithelial cells and activates neutrophils via Toll-like receptor (TLR)-4. Respir. Res..

[62] Borde C., Barnay-Verdier S., Gaillard C., Hocini H., Marechal V., Gozlan J. (2011). Stepwise release of biologically active HMGB1 during HSV-2 infection. PLoS ONE.

[63] Turville S.G., Santos J.J., Frank I. (2004). Immunodeficiency virus uptake, turnover, and 2-phase transfer in human dendritic cells. Blood.

[64] Geijtenbeek T.B., Kwon D.S., Torensma R. (2000). DC-SIGN, a dendritic cell-specific HIV-1-binding protein that enhances trans-infection of T cells. Cell.

[65] Arrighi J.F., Pion M., Garcia E. (2004). DC-SIGN-mediated infectious synapse formation enhances X4 HIV-1 transmission from dendritic cells to T cells. J. Exp. Med..

[66] Hodges A., Sharrocks K., Edelmann M. (2007). Activation of the lectin DC-SIGN induces an immature dendritic cell phenotype triggering Rho-GTPase activity required for HIV-1 replication. Nat. Immunol..

[67] Lai J., Bernhard O.K., Turville S.G., Harman A.N., Wilkinson J., Cunningham A.L. (2009). Oligomerization of the macrophage mannose receptor enhances gp120-mediated binding of HIV-1. J. Biol. Chem..

[68] Gringhuis S.I., van d V., van den Berg L.M., den Dunnen J., Litjens M., Geijtenbeek T.B. (2010). HIV-1 exploits innate signaling by TLR8 and DC-SIGN for productive infection of dendritic cells. Nat. Immunol..

[69] Tassaneetrithep B., Burgess T.H., Granelli-Piperno A. (2003). DC-SIGN (CD209) mediates dengue virus infection of human dendritic cells. J. Exp. Med..

[70] Alvarez C.P., Lasala F., Carrillo J., Muniz O., Corbi A.L., Delgado R. (2002). C-type lectins DC-SIGN and L-SIGN mediate cellular entry by Ebola virus in *cis* and in *trans*. J. Virol..

[71] Halary F., Amara A., Lortat-Jacob H. (2002). Human cytomegalovirus binding to DC-SIGN is required for dendritic cell infection and target cell trans-infection. Immunity.

[72] Geijtenbeek T.B., Gringhuis S.I. (2009). Signalling through C-type lectin receptors: shaping immune responses. Nat. Rev. Immunol..

[73] Holm C.K., Jensen S.B., Jakobsen M.R. (2012). Virus-cell fusion as a trigger of innate immunity dependent on the adaptor STING. Nat. Immunol..

[74] Soby S., Laursen R.R., Ostergaard L., Melchjorsen J. (2012). HSV-1-induced chemokine expression via IFI16-dependent and IFI16-independent pathways in human monocyte-derived macrophages. Herpesviridae.

[75] Barlan A.U., Griffin T.M., McGuire K.A., Wiethoff C.M. (2011). Adenovirus membrane penetration activates the NLRP3 inflammasome. J. Virol..

[76] Weber F., Wagner V., Rasmussen S.B., Hartmann R., Paludan S.R. (2006). Double-stranded RNA is produced by positive-strand RNA viruses and DNA viruses but not in detectable amounts by negative-strand RNA viruses. J. Virol..

[77] Kato H., Takeuchi O., Mikamo-Satoh E. (2008). Length-dependent recognition of double-stranded ribonucleic acids by retinoic acid-inducible gene-I and melanoma differentiation-associated gene 5. J. Exp. Med..

[78] Reinert L.S., Harder L., Holm C.K. (2012). TLR3 deficiency renders astrocytes permissive to herpes simplex virus infection and facilitates establishment of CNS infection in mice. J. Clin. Invest..

[79] Hutchens M., Luker K.E., Sottile P. (2008). TLR3 increases disease morbidity and mortality from vaccinia infection. J. Immunol..

[80] Schulz O., Diebold S.S., Chen M. (2005). Toll-like receptor 3 promotes cross-priming to virus-infected cells. Nature.

[81] Wang J.P., Liu P., Latz E., Golenbock D.T., Finberg R.W., Libraty D.H. (2006). Flavivirus activation of plasmacytoid dendritic cells delineates key elements of TLR7 signaling beyond endosomal recognition. J. Immunol..

[82] Martinez J., Huang X., Yang Y. (2010). Toll-like receptor 8-mediated activation of murine plasmacytoid dendritic cells by vaccinia viral DNA. Proc. Natl. Acad. Sci. U.S.A..

[83] Bauer S., Bathke B., Lauterbach H. (2010). A major role for TLR8 in the recognition of vaccinia viral DNA by murine pDC?. Proc. Natl. Acad. Sci. U.S.A..

[84] Lepelley A., Louis S., Sourisseau M. (2011). Innate Sensing of HIV-Infected Cells. PLoS Pathog.

[85] Wagner H. (2004). The immunobiology of the TLR9 subfamily. Trends Immunol..

[86] Hochrein H., Schlatter B. (2004). Herpes simplex virus type-1 induces IFN-alpha production via Toll-like receptor 9-dependent and -independent pathways. Proc. Natl. Acad. Sci. U.S.A..

[87] Megjugorac N.J., Young H.A., Amrute S.B., Olshalsky S.L., Fitzgerald-Bocarsly P. (2004). Virally stimulated plasmacytoid dendritic cells produce chemokines and induce migration of T and NK cells. J. Leukoc. Biol..

[88] Megjugorac N.J., Gallagher G.E., Gallagher G. (2009). Modulation of human plasmacytoid DC function by IFN-lambda1 (IL-29). J. Leukoc. Biol..

[89] Lee H.K., Lund J.M., Ramanathan B., Mizushima N., Iwasaki A. (2007). Autophagy-dependent viral recognition by plasmacytoid dendritic cells. Science.

[90] Rehwinkel J., Tan C.P., Goubau D. (2010). RIG-I detects viral genomic RNA during negative-strand RNA virus infection. Cell.

[91] Loo Y.M., Fornek J., Crochet N. (2008). Distinct RIG-I and MDA5 signaling by RNA viruses in innate immunity. J. Virol..

[92] Kato H., Takeuchi O., Sato S. (2006). Differential roles of MDA5 and RIG-I helicases in the recognition of RNA viruses. Nature.

[93] Solis M., Nakhaei P., Jalalirad M. (2011). RIG-I-mediated antiviral signaling is inhibited in HIV-1 infection by a protease-mediated sequestration of RIG-I. J. Virol..

[94] Berg R.K., Melchjorsen J., Rintahaka J. (2012). Genomic HIV RNA Induces Innate Immune Responses through RIG-I-Dependent Sensing of Secondary-Structured RNA. PLoS ONE.

[95] Samanta M., Iwakiri D., Kanda T., Imaizumi T., Takada K. (2006). EB virus-encoded RNAs are recognized by RIG-I and activate signaling to induce type I IFN. EMBO. J..

[96] Holm G.H., Zurney J., Tumilasci V. (2007). Retinoic acid-inducible gene-I and interferon-beta promoter stimulator-1 augment proapoptotic responses following mammalian reovirus infection via interferon regulatory factor-3. J. Biol. Chem..

[97] Sen A., Pruijssers A.J., Dermody T.S., Garcia-Sastre A., Greenberg H.B. (2011). The early interferon response to rotavirus is regulated by PKR and depends on MAVS/IPS-1, RIG-I, MDA-5, and IRF3. J. Virol..

[98] Myskiw C., Arsenio J., Booy E.P. (2011). RNA species generated in vaccinia virus infected cells activate cell type-specific MDA5 or RIG-I dependent interferon gene transcription and PKR dependent apoptosis. Virology.

[99] Ikegame S., Takeda M., Ohno S., Nakatsu Y., Nakanishi Y., Yanagi Y. (2010). Both RIG-I and MDA5 RNA helicases contribute to the induction of alpha/beta interferon in measles virus-infected human cells. J. Virol..

[100] Liu P., Jamaluddin M., Li K., Garofalo R.P., Casola A., Brasier A.R. (2007). Retinoic acid-inducible gene I mediates early antiviral response and Toll-like receptor 3 expression in respiratory syncytial virus-infected airway epithelial cells. J. Virol..

[101] Kato H., Sato S., Yoneyama M. (2005). Cell Type-Specific Involvement of RIG-I in Antiviral Response. Immunity.

[102] Sabbah A., Bose S. (2009). Retinoic acid inducible gene I activates innate antiviral response against human parainfluenza virus type 3. Virol. J..

[103] Poeck H., Bscheider M., Gross O. (2010). Recognition of RNA virus by RIG-I results in activation of CARD9 and inflammasome signaling for interleukin 1 beta production. Nat. Immunol..

[104] Melchjorsen J., Rintahaka J., Soby S. (2010). Early innate recognition of herpes simplex virus in human primary macrophages is mediated via the MDA5/MAVS-dependent and MDA5/MAVS/RNA polymerase III-independent pathways. J. Virol..

[105] Pichlmair A., Schulz O., Tan C.P. (2009). Activation of MDA5 requires higher-order RNA structures generated during virus infection. J. Virol..

[106] Wang J.P., Cerny A., Asher D.R., Kurt-Jones E.A., Bronson R.T., Finberg R.W. (2010). MDA5 and MAVS mediate type I interferon responses to coxsackie B virus. J. Virol..

[107] Yount J.S., Gitlin L., Moran T.M., Lopez C.B. (2008). MDA5 Participates in the Detection of Paramyxovirus Infection and Is Essential for the Early Activation of Dendritic Cells in Response to Sendai Virus Defective Interfering Particles. J. Immunol..

[108] Miyashita M., Oshiumi H., Matsumoto M., Seya T. (2011). DDX60, a DExD/H box helicase, is a novel antiviral factor promoting RIG-I-like receptor-mediated signaling. Mol. Cell. Biol..

[109] Zhang Z., Yuan B., Lu N., Facchinetti V., Liu Y.J. (2011). DHX9 pairs with IPS-1 to sense double-stranded RNA in myeloid dendritic cells. J. Immunol..

[110] Zhang Z., Kim T., Bao M. (2011). DDX1, DDX21, and DHX36 Helicases Form a Complex with the Adaptor Molecule TRIF to Sense dsRNA in Dendritic Cells. Immunity.

[111] Sabbah A., Chang T.H., Harnack R. (2009). Activation of innate immune antiviral responses by Nod2. Nat. Immunol..

[112] Kanneganti T.D., Body-Malapel M., Amer A. (2006). Critical role for Cryopyrin/Nalp3 in activation of caspase-1 in response to viral infection and double-stranded RNA. J. Biol. Chem..

[113] Allen I.C., Scull M.A., Moore C.B. (2009). The NLRP3 inflammasome mediates in vivo innate immunity to influenza A virus through recognition of viral RNA. Immunity.

[114] Ichinohe T., Pang I.K., Iwasaki A. (2010). Influenza virus activates inflammasomes via its intracellular M2 ion channel. Nat. Immunol..

[115] Thomas P.G., Dash P., Aldridge J.R. (2009). The intracellular sensor NLRP3 mediates key innate and healing responses to influenza A virus via the regulation of caspase-1. Immunity.

[116] Rajan J.V., Rodriguez D., Miao E.A., Aderem A. (2011). The NLRP3 inflammasome detects encephalomyocarditis virus and vesicular stomatitis virus infection. J. Virol..

[117] Muruve D.A., Petrilli V., Zaiss A.K. (2008). The inflammasome recognizes cytosolic microbial and host DNA and triggers an innate immune response. Nature.

[118] Nour A.M., Reichelt M., Ku C.C., Ho M.Y., Heineman T.C., Arvin A.M. (2011). Varicella-zoster virus infection triggers formation of an interleukin-1beta (IL-1beta)-processing inflammasome complex. J. Biol. Chem..

[119] Melchjorsen J., Pedersen F.S., Mogensen S.C., Paludan S.R. (2002). Herpes simplex virus selectively induces expression of the CC Chemokine RANTES/CCL5 in macrophages through a mechanism dependent on PKR and ICP0. J. Virol..

[120] Yang P., An H., Liu X. (2010). The cytosolic nucleic acid sensor LRRFIP1 mediates the production of type I interferon via a beta-catenin-dependent pathway. Nat. Immunol..

[121] Chiu Y.H., Macmillan J.B., Chen Z.J. (2009). RNA Polymerase III Detects Cytosolic DNA and Induces Type I Interferons through the RIG-I Pathway. Cell.

[122] Ablasser A., Bauernfeind F., Hartmann G., Latz E., Fitzgerald K.A., Hornung V. (2009). RIG-I-dependent sensing of poly(dA:dT) through the induction of an RNA polymerase III-transcribed RNA intermediate. Nat. Immunol..

[123] Unterholzner L., Keating S.E., Baran M. (2010). IFI16 is an innate immune sensor for intracellular DNA. Nat. Immunol..

[124] Takaoka A., Wang Z., Choi M.K. (2007). DAI (DLM-1/ZBP1) is a cytosolic DNA sensor and an activator of innate immune response. Nature.

[125] DeFilippis V.R., Alvarado D., Sali T., Rothenburg S., Fruh K. (2009). Human Cytomegalovirus Induces the Interferon Response Via the DNA Sensor ZBP1. J. Virol..

[126] Kim T., Pazhoor S., Bao M. (2010). Aspartate-glutamate-alanine-histidine box motif (DEAH)/RNA helicase A helicases sense microbial DNA in human plasmacytoid dendritic cells. Proc. Natl. Acad. Sci. U.S.A..

[127] Zhang Z., Yuan B., Bao M., Lu N., Kim T., Liu Y.J. (2011). The helicase DDX41 senses intracellular DNA mediated by the adaptor STING in dendritic cells. Nat. Immunol..

[128] Zhang X., Brann T.W., Zhou M. (2011). Cutting edge: Ku70 is a novel cytosolic DNA sensor that induces type III rather than type I IFN. J. Immunol..

[129] Rathinam V.A., Jiang Z., Waggoner S.N. (2010). The AIM2 inflammasome is essential for host defense against cytosolic bacteria and DNA viruses. Nat. Immunol..

[130] Kerur N., Veettil M.V., Sharma-Walia N. (2011). IFI16 Acts as a Nuclear Pathogen Sensor to Induce the Inflammasome in Response to Kaposi Sarcoma-Associated Herpesvirus Infection. Cell Host Microbe.

[131] Manel N., Hogstad B., Wang Y., Levy D.E., Unutmaz D., Littman D.R. (2010). A cryptic sensor for HIV-1 activates antiviral innate immunity in dendritic cells. Nature.

[132] Pertel T., Hausmann S., Morger D. (2011). TRIM5 is an innate immune sensor for the retrovirus capsid lattice. Nature.

[133] Pichlmair A., Lassnig C., Eberle C.A. (2011). IFIT1 is an antiviral protein that recognizes 5'-triphosphate RNA. Nat. Immunol..

[134] Li K., Chen Z., Kato N., Gale M., Lemon S.M. (2005). Distinct poly-I: C and virus-activated signaling pathways leading to interferon-beta production in hepatocytes. J. Biol. Chem..

[135] Seth R.B., Sun L., Ea C.K., Chen Z.J. (2005). Identification and Characterization of MAVS, a Mitochondrial Antiviral Signaling Protein that Activates NF-kappaB and IRF3. Cell.

[136] Xu L.G., Wang Y.Y., Han K.J., Li L.Y., Zhai Z., Shu H.B. (2005). VISA Is an Adapter Protein Required for Virus-Triggered IFN-beta Signaling. Mol. Cell..

[137] Kawai T., Takahashi K., Sato S. (2005). IPS-1, an adaptor triggering RIG-I- and Mda5-mediated type I interferon induction. Nat. Immunol..

[138] Meylan E., Curran J., Hofmann K. (2005). Cardif is an adaptor protein in the RIG-I antiviral pathway and is targeted by hepatitis C virus. Nature.

[139] Venkataraman T., Valdes M., Elsby R. (2007). Loss of DExD/H box RNA helicase LGP2 manifests disparate antiviral responses. J. Immunol..

[140] Yoneyama M., Kikuchi M., Matsumoto K. (2005). Shared and Unique Functions of the DExD/H-Box Helicases RIG-I, MDA5, and LGP2 in Antiviral Innate Immunity. J. Immunol..

[141] Rothenfusser S., Goutagny N., DiPerna G. (2005). The RNA Helicase Lgp2 Inhibits TLR-Independent Sensing of Viral Replication by Retinoic Acid-Inducible Gene-I. J. Immunol..

[142] Satoh T., Kato H., Kumagai Y. (2010). LGP2 is a positive regulator of RIG-I- and MDA5-mediated antiviral responses. Proc. Natl. Acad. Sci. U.S.A..

[143] Broquet A.H., Hirata Y., McAllister C.S., Kagnoff M.F. (2011). RIG-I/MDA5/MAVS are required to signal a protective IFN response in rotavirus-infected intestinal epithelium. J. Immunol..

[144] Burdette D., Haskett A., Presser L., McRae S., Iqbal J., Waris G. (2012). Hepatitis C virus activates interleukin-1beta via caspase-1-inflammasome complex. J. Gen. Virol..

[145] Segovia J., Sabbah A., Mgbemena V. (2012). TLR2/MyD88/NF-kappaB pathway, reactive oxygen species, potassium efflux activates NLRP3/ASC inflammasome during respiratory syncytial virus infection. PLoS ONE.

[146] Pontillo A., Silva L.T., Oshiro T.M., Finazzo C., Crovella S., Duarte A.J. (2012). HIV-1 induces NALP3-inflammasome expression and interleukin-1beta secretion in dendritic cells from healthy individuals but not from HIV-positive patients. AIDS.

[147] Pontillo A., Brandao L.A., Guimaraes R.L., Segat L., Athanasakis E., Crovella S. (2010). A 3'UTR SNP in NLRP3 gene is associated with susceptibility to HIV-1 infection. J. Acquir. Immune. Defic. Syndr..

[148] Pontillo A., Oshiro T.M., Girardelli M., Kamada A.J., Crovella S., Duarte A.J. (2012). Polymorphisms in inflammasome' genes and susceptibility to HIV-1 infection. J. Acquir. Immune. Defic. Syndr..

[149] Suzuki K., Mori A., Ishii K.J. (1999). Activation of target-tissue immune-recognition molecules by double-stranded polynucleotides. Proc. Natl. Acad. Sci. U.S.A..

[150] Doitsh G., Cavrois M., Lassen K.G. (2010). Abortive HIV infection mediates CD4 T cell depletion and inflammation in human lymphoid tissue. Cell.

[151] Furr S.R., Chauhan V.S., Moerdyk-Schauwecker M.J., Marriott I. (2011). A role for DNA-dependent activator of interferon regulatory factor in the recognition of herpes simplex virus type 1 by glial cells. J. Neuroinflammation.

[152] Hayashi T., Nishitsuji H., Takamori A., Hasegawa A., Masuda T., Kannagi M. (2010). DNA-dependent activator of IFN-regulatory factors enhances the transcription of HIV-1 through NF-kappaB. Microbes. Infect..

[153] Downs J.A., Jackson S.P. (2004). A means to a DNA end: the many roles of Ku. Nat. Rev. Mol. Cell. Biol..

[154] Stein S.C., Falck-Pedersen E. (2012). Sensing adenovirus infection: activation of interferon regulatory factor 3 in RAW 264.7 cells. J. Virol..

[155] Veeranki S., Duan X., Panchanathan R., Liu H., Choubey D. (2011). IFI16 Protein Mediates the Anti-inflammatory Actions of the Type-I Interferons through Suppression of Activation of Caspase-1 by Inflammasomes. PLoS ONE.

[156] Gariano G.R., Dell'Oste V., Bronzini M. (2012). The intracellular DNA sensor IFI16 gene acts as restriction factor for human cytomegalovirus replication. PLoS Pathog.

[157] Conrady C.D., Zheng M., Fitzgerald K.A., Liu C., Carr D.J. (2012). Resistance to HSV-1 infection in the epithelium resides with the novel innate sensor, IFI-16. Mucosal. Immunol..

[158] Kis-Toth K., Szanto A., Thai T.H., Tsokos G.C. (2011). Cytosolic DNA-Activated Human Dendritic Cells Are Potent Activators of the Adaptive Immune Response. J. Immunol..

[159] Yamaguchi T., Kawabata K., Kouyama E. (2010). Induction of type I interferon by adenovirus-encoded small RNAs. Proc. Natl. Acad. Sci. U.S.A..

[160] Dai P., Jeong S.Y., Yu Y. (2009). Modulation of TLR signaling by multiple MyD88-interacting partners including leucine-rich repeat Fli-I-interacting proteins. J. Immunol..

[161] Hornung V., Ablasser A., Charrel-Dennis M. (2009). AIM2 recognizes cytosolic dsDNA and forms a caspase-1-activating inflammasome with ASC. Nature.

[162] Fernandes-Alnemri T., Yu J.W., Datta P., Wu J., Alnemri E.S. (2009). AIM2 activates the inflammasome and cell death in response to cytoplasmic DNA. Nature.

[163] Chintakuntlawar A.V., Zhou X., Rajaiya J., Chodosh J. (2010). Viral capsid is a pathogen-associated molecular pattern in adenovirus keratitis. PLoS Pathog.

[164] Williams B.R. (2001). Signal integration via PKR. Sci. STKE.

[165] Nallagatla S.R., Toroney R., Bevilacqua P.C. (2011). Regulation of innate immunity through RNA structure and the protein kinase PKR. Curr. Opin. Struct. Biol..

[166] Zamanian-Daryoush M., Mogensen T.H., DiDonato J.A., Williams B.R. (2000). NF-kappaB activation by double-stranded-RNA-activated protein kinase (PKR) is mediated through NF-kappaB-inducing kinase and IkappaB kinase. Mol. Cell. Biol..

[167] Nallagatla S.R., Hwang J., Toroney R., Zheng X., Cameron C.E., Bevilacqua P.C. (2007). 5'-triphosphate-dependent activation of PKR by RNAs with short stem-loops. Science.

[168] Kim I., Liu C.W., Puglisi J.D. (2006). Specific recognition of HIV TAR RNA by the dsRNA binding domains (dsRBD1-dsRBD2) of PKR. J. Mol. Biol..

[169] Arnaud N., Dabo S., Maillard P. (2010). Hepatitis C virus controls interferon production through PKR activation. PLoS ONE.

[170] Talloczy Z., Virgin H.W., Levine B. (2006). PKR-dependent autophagic degradation of herpes simplex virus type 1. Autophagy.

[171] English L., Chemali M., Duron J. (2009). Autophagy enhances the presentation of endogenous viral antigens on MHC class I molecules during HSV-1 infection. Nat. Immunol..

[172] McAllister C.S., Samuel C.E. (2009). The RNA-activated protein kinase enhances the induction of interferon-beta and apoptosis mediated by cytoplasmic RNA sensors. J. Biol. Chem..

[173] Wang H., Bloom O., Zhang M. (1999). HMG-1 as a late mediator of endotoxin lethality in mice. Science.

[174] Yanai H., Ban T., Wang Z. (2009). HMGB proteins function as universal sentinels for nucleic-acid-mediated innate immune responses. Nature.

[175] Moisy D., Avilov S.V., Jacob Y. (2012). HMGB1 protein binds to influenza virus nucleoprotein and promotes viral replication. J. Virol..

[176] Matsumoto Y., Hayashi Y., Omori H. (2012). Bornavirus closely associates and segregates with host chromosomes to ensure persistent intranuclear infection. Cell Host Microbe.

[177] Saidi H., Melki M.T., Gougeon M.L. (2008). HMGB1-dependent triggering of HIV-1 replication and persistence in dendritic cells as a consequence of NK-DC cross-talk. PLoS ONE.

[178] Thierry S., Gozlan J., Jaulmes A. (2007). High-mobility group box 1 protein induces HIV-1 expression from persistently infected cells. AIDS.

[179] Cassetta L., Fortunato O., Adduce L. (2009). Extracellular high mobility group box-1 inhibits R5 and X4 HIV-1 strains replication in mononuclear phagocytes without induction of chemokines and cytokines. AIDS.

[180] Barqasho B., Nowak P., Abdurahman S., Walther-Jallow L., Sonnerborg A. (2010). Implications of the release of high-mobility group box 1 protein from dying cells during human immunodeficiency virus type 1 infection *in vitro*. J. Gen. Virol..

[181] Nowak P., Barqasho B., Sonnerborg A. (2007). Elevated plasma levels of high mobility group box protein 1 in patients with HIV-1 infection. AIDS.

[182] Troseid M., Nowak P., Nystrom J., Lindkvist A., Abdurahman S., Sonnerborg A. (2010). Elevated plasma levels of lipopolysaccharide and high mobility group box-1 protein are associated with high viral load in HIV-1 infection: reduction by 2-year antiretroviral therapy. AIDS.

[183] Yoneyama M., Fujita T. (2010). Recognition of viral nucleic acids in innate immunity. Rev. Med. Virol..

[184] Rathinam V.A., Fitzgerald K.A. (2011). Innate immune sensing of DNA viruses. Virology.

[185] Kim M.J., Hwang S.Y., Imaizumi T., Yoo J.Y. (2008). Negative feedback regulation of RIG-I-mediated antiviral signaling by interferon-induced ISG15 conjugation. J. Virol..

[186] Lin R., Yang L., Nakhaei P. (2006). Negative regulation of the retinoic acid-inducible gene I-induced antiviral state by the ubiquitin-editing protein A20. J. Biol. Chem..

[187] Ishikawa H., Ma Z., Barber G.N. (2009). STING regulates intracellular DNA-mediated, type I interferon-dependent innate immunity. Nature.

[188] Ishikawa H., Barber G.N. (2008). STING is an endoplasmic reticulum adaptor that facilitates innate immune signalling. Nature.

[189] Parker D., Martin F.J., Soong G. (2011). Streptococcus pneumoniae DNA initiates type I interferon signaling in the respiratory tract. mBio.

[190] Taniguchi T., Ogasawara K., Takaoka A., Tanaka N. (2001). IRF family of transcription factors as regulators of host defense. Annu. Rev. Immunol..

[191] Tsuruta L., Arai N., Arai K. (1998). Transcriptional control of cytokine genes. Int. Rev. Immunol..

[192] Melchjorsen J., Sorensen L.N., Paludan S.R. (2003). Expression and function of chemokines during viral infections: from molecular mechanisms to in vivo function. J. Leukoc. Biol..

[193] Melchjorsen J., Paludan S.R. (2003). Induction of RANTES/CCL5 by herpes simplex virus is regulated by NF-kappaB and IRF3. J. Gen. Virol..

[194] Melchjorsen J., Kristiansen H., Christiansen R. (2009). Differential Regulation of the OASL and OAS1 Genes in Response to Viral Infections. J. Interferon Cytokine Res..

[195] Leonard W.J., O'Shea J.J. (1998). Jaks and STATs: Biological implications. Annu. Rev. Immunol..

[196] Der S.D., Zhou A., Williams B.R., Silverman R.H. (1998). Identification of genes differentially regulated by interferon alpha, beta, or gamma using oligonucleotide arrays. Proc. Natl. Acad. Sci. U.S.A..

[197] Sadler A.J., Williams B.R. (2008). Interferon-inducible antiviral effectors. Nat. Rev. Immunol..

[198] Kotenko S.V., Gallagher G., Baurin V.V. (2003). IFN-lambdas mediate antiviral protection through a distinct class II cytokine receptor complex. Nat. Immunol..

[199] Sheppard P., Kindsvogel W., Xu W. (2003). IL-28, IL-29 and their class II cytokine receptor IL-28R. Nat. Immunol..

[200] Meager A., Visvalingam K., Dilger P., Bryan D., Wadhwa M. (2005). Biological activity of interleukins-28 and -29: Comparison with type I interferons. Cytokine.

[201] Dellgren C., Gad H.H., Hamming O.J., Melchjorsen J., Hartmann R. (2009). Human interferon-lambda3 is a potent member of the type III interferon family. Genes Immun..

[202] Ank N., West H., Bartholdy C., Eriksson K., Thomsen A.R., Paludan S.R. (2006). Lambda Interferon (IFN-{lambda}), a Type III IFN, Is Induced by Viruses and IFNs and Displays Potent Antiviral Activity against Select Virus Infections In Vivo. J. Virol..

[203] Osterlund P., Veckman V., Siren J. (2005). Gene expression and antiviral activity of alpha/beta interferons and interleukin-29 in virus-infected human myeloid dendritic cells. J. Virol..

[204] Melchjorsen J., Siren J., Julkunen I., Paludan S.R., Matikainen S. (2006). Induction of cytokine expression by herpes simplex virus in human monocyte-derived macrophages and dendritic cells is dependent on virus replication and is counteracted by ICP27 targeting NF-kappaB and IRF-3. J. Gen. Virol..

[205] Berghall H., Siren J., Sarkar D. (2006). The interferon-inducible RNA helicase, mda-5, is involved in measles virus-induced expression of antiviral cytokines. Microbes Infect..

[206] Coccia E.M., Severa M., Giacomini E. (2004). Viral infection and Toll-like receptor agonists induce a differential expression of type I and lambda interferons in human plasmacytoid and monocyte-derived dendritic cells. Eur. J. Immunol..

[207] Hou W., Wang X., Ye L. (2009). Lambda interferon inhibits human immunodeficiency virus type 1 infection of macrophages. J. Virol..

[208] Robek M.D., Boyd B.S., Chisari F.V. (2005). Lambda interferon inhibits hepatitis B and C virus replication. J. Virol..

[209] Sommereyns C., Paul S., Staeheli P., Michiels T. (2008). IFN-lambda (IFN-lambda) is expressed in a tissue-dependent fashion and primarily acts on epithelial cells in vivo. PLoS Pathog.

[210] Ank N., Iversen M.B., Bartholdy C. (2008). An important role for type III Interferon (IFN-lambda/IL-28) in TLR-induced antiviral activity. J. Immunol..

[211] Kerr I.M., Brown R.E., Hovanessian A.G. (1977). Nature of inhibitor of cell-free protein synthesis formed in response to interferon and double-stranded RNA. Nature.

[212] Floyd-Smith G., Slattery E., Lengyel P. (1981). Interferon action: RNA cleavage pattern of a (2'-5')oligoadenylate--dependent endonuclease. Science.

[213] Hovanessian A.G. (1991). Interferon-induced and double-stranded RNA-activated enzymes: A specific protein kinase and 2',5'-oligoadenylate synthetases. J. Interferon. Res..

[214] Rebouillat D., Marie I., Hovanessian A.G. (1998). Molecular cloning and characterization of two related and interferon-induced 56-kDa and 30-kDa proteins highly similar to 2'-5' oligoadenylate synthetase. Eur. J. Biochem..

[215] Sharp T.V., Raine D.A., Gewert D.R., Joshi B., Jagus R., Clemens M.J. (1999). Activation of the interferon-inducible (2'-5') oligoadenylate synthetase by the Epstein-Barr virus RNA, EBER-1. Virology.

[216] Desai S.Y., Patel R.C., Sen G.C., Malhotra P., Ghadge G.D., Thimmapaya B. (1995). Activation of interferon-inducible 2'-5' oligoadenylate synthetase by adenoviral VAI RNA. J. Biol. Chem..

[217] Maitra R.K., McMillan N.A., Desai S. (1994). HIV-1 TAR RNA has an intrinsic ability to activate interferon-inducible enzymes. Virology.

[218] Yakub I., Lillibridge K.M., Moran A. (2005). Single nucleotide polymorphisms in genes for 2'-5'-oligoadenylate synthetase and RNase L inpatients hospitalized with West Nile virus infection. J. Infect. Dis..

[219] Lim J.K., Lisco A., McDermott D.H. (2009). Genetic variation in OAS1 is a risk factor for initial infection with West Nile virus in man. PLoS Pathog.

[220] Rios J.J., Fleming J.G., Bryant U.K. (2010). OAS1 polymorphisms are associated with susceptibility to West Nile encephalitis in horses. PLoS ONE.

[221] Knapp S., Yee L.J., Frodsham A.J. (2003). Polymorphisms in interferon-induced genes and the outcome of hepatitis C virus infection: roles of MxA, OAS-1 and PKR. Genes Immun..

[222] El Awady M.K., Anany M.A., Esmat G. (2011). Single nucleotide polymorphism at exon 7 splice acceptor site of OAS1 gene determines response of hepatitis C virus patients to interferon therapy. J. Gastroenterol. Hepatol..

[223] Haralambieva I.H., Dhiman N., Ovsyannikova I.G. (2010). 2'-5'-Oligoadenylate synthetase single-nucleotide polymorphisms and haplotypes are associated with variations in immune responses to rubella vaccine. Hum. Immunol..

[224] Kristiansen H., Scherer C.A., McVean M. (2010). Extracellular 2'-5' oligoadenylate synthetase stimulates RNase L-independent antiviral activity: a novel mechanism of virus-induced innate immunity. J. Virol..

[225] Malathi K., Dong B., Gale M., Silverman R.H. (2007). Small self-RNA generated by RNase L amplifies antiviral innate immunity. Nature.

[226] Lenschow D.J., Lai C., Frias-Staheli N. (2007). IFN-stimulated gene 15 functions as a critical antiviral molecule against influenza, herpes, and Sindbis viruses. Proc. Natl. Acad. Sci. U.S.A..

[227] Pincetic A., Kuang Z., Seo E.J., Leis J. (2010). The interferon-induced gene ISG15 blocks retrovirus release from cells late in the budding process. J. Virol..

[228] Kuang Z., Seo E.J., Leis J. (2011). Mechanism of Inhibition of Retrovirus Release from Cells by Interferon-Induced Gene ISG15. J. Virol..

[229] Zhao C., Hsiang T.Y., Kuo R.L., Krug R.M. (2010). ISG15 conjugation system targets the viral NS1 protein in influenza A virus-infected cells. Proc. Natl. Acad. Sci. U.S.A..

[230] Dai J., Pan W., Wang P. (2011). ISG15 facilitates cellular antiviral response to dengue and west nile virus infection *in vitro*. Virol. J..

[231] Okumura A., Lu G., Pitha-Rowe I., Pitha P.M. (2006). Innate antiviral response targets HIV-1 release by the induction of ubiquitin-like protein ISG15. Proc. Natl. Acad. Sci. U.S.A..

[232] Lu G., Reinert J.T., Pitha-Rowe I. (2006). ISG15 enhances the innate antiviral response by inhibition of IRF-3 degradation. Cell Mol. Biol..

[233] Osiak A., Utermohlen O., Niendorf S., Horak I., Knobeloch K.P. (2005). ISG15, an interferon-stimulated ubiquitin-like protein, is not essential for STAT1 signaling and responses against vesicular stomatitis and lymphocytic choriomeningitis virus. Mol. Cell. Biol..

[234] Chieux V., Chehadeh W., Harvey J., Haller O., Wattre P., Hober D. (2001). Inhibition of coxsackievirus B4 replication in stably transfected cells expressing human MxA protein. Virology.

[235] Gordien E., Rosmorduc O., Peltekian C., Garreau F., Brechot C., Kremsdorf D. (2001). Inhibition of hepatitis B virus replication by the interferon-inducible MxA protein. J. Virol..

[236] Turan K., Mibayashi M., Sugiyama K., Saito S., Numajiri A., Nagata K. (2004). Nuclear MxA proteins form a complex with influenza virus NP and inhibit the transcription of the engineered influenza virus genome. Nucleic. Acids. Res..

[237] Kochs G., Haller O. (1999). Interferon-induced human MxA GTPase blocks nuclear import of Thogoto virus nucleocapsids. Proc. Natl. Acad. Sci. U.S.A..

[238] Kochs G., Janzen C., Hohenberg H., Haller O. (2002). Antivirally active MxA protein sequesters La Crosse virus nucleocapsid protein into perinuclear complexes. Proc. Natl. Acad. Sci. U.S.A..

[239] Hijikata M., Ohta Y., Mishiro S. (2000). Identification of a single nucleotide polymorphism in the MxA gene promoter (G/T at nt -88) correlated with the response of hepatitis C patients to interferon. Intervirology.

[240] Suzuki F., Arase Y., Suzuki Y. (2004). Single nucleotide polymorphism of the MxA gene promoter influences the response to interferon monotherapy in patients with hepatitis C viral infection. J. Viral. Hepat..

[241] Chin K.C., Cresswell P. (2001). Viperin (cig5), an IFN-inducible antiviral protein directly induced by human cytomegalovirus. Proc. Natl. Acad. Sci. U.S.A..

[242] Wang X., Hinson E.R., Cresswell P. (2007). The interferon-inducible protein viperin inhibits influenza virus release by perturbing lipid rafts. Cell Host Microbe..

[243] Helbig K.J., Eyre N.S., Yip E. (2011). The antiviral protein viperin inhibits hepatitis C virus replication via interaction with nonstructural protein 5A. Hepatology.

[244] Wang S., Wu X., Pan T. (2012). Viperin inhibits hepatitis C virus replication by interfering with binding of NS5A to host protein hVAP-33. J. Gen. Virol..

[245] Szretter K.J., Brien J.D., Thackray L.B., Virgin H.W., Cresswell P., Diamond M.S. (2011). The interferon-inducible gene viperin restricts West Nile virus pathogenesis. J. Virol..

[246] Lim E.S., Wu L.I., Malik H.S., Emerman M. (2012). The function and evolution of the restriction factor viperin in primates was not driven by lentiviruses. Retrovirology.

[247] Saitoh T., Satoh T., Yamamoto N. (2011). Antiviral protein Viperin promotes Toll-like receptor 7- and Toll-like receptor 9-mediated type I interferon production in plasmacytoid dendritic cells. Immunity.

[248] Fensterl V., Wetzel J.L., Ramachandran S. (2012). Interferon-induced Ifit2/ISG54 protects mice from lethal VSV neuropathogenesis. PLoS Pathog.

[249] Harris R.S., Liddament M.T. (2004). Retroviral restriction by APOBEC proteins. Nat. Rev. Immunol..

[250] Mangeat B., Turelli P., Caron G., Friedli M., Perrin L., Trono D. (2003). Broad antiretroviral defence by human APOBEC3G through lethal editing of nascent reverse transcripts. Nature.

[251] Bishop K.N., Holmes R.K., Malim M.H. (2006). Antiviral potency of APOBEC proteins does not correlate with cytidine deamination. J. Virol..

[252] Holmes R.K., Koning F.A., Bishop K.N., Malim M.H. (2007). APOBEC3F can inhibit the accumulation of HIV-1 reverse transcription products in the absence of hypermutation. Comparisons with APOBEC3G. J. Biol. Chem..

[253] Wang F.X., Huang J., Zhang H., Ma X., Zhang H. (2008). APOBEC3G upregulation by alpha interferon restricts human immunodeficiency virus type 1 infection in human peripheral plasmacytoid dendritic cells. J. Gen. Virol..

[254] Argyris E.G., Acheampong E., Wang F. (2007). The interferon-induced expression of APOBEC3G in human blood-brain barrier exerts a potent intrinsic immunity to block HIV-1 entry to central nervous system. Virology.

[255] Suspene R., Aynaud M.M., Koch S. (2011). Genetic Editing of Herpes Simplex Virus 1 and Epstein-Barr Herpesvirus Genomes by Human APOBEC3 Cytidine Deaminases in Culture and *In Vivo*. J. Virol..

[256] Vartanian J.P., Guetard D., Henry M., Wain-Hobson S. (2008). Evidence for editing of human papillomavirus DNA by APOBEC3 in benign and precancerous lesions. Science.

[257] Turelli P., Mangeat B., Jost S., Vianin S., Trono D. (2004). Inhibition of hepatitis B virus replication by APOBEC3G. Science.

[258] Suspene R., Guetard D., Henry M., Sommer P., Wain-Hobson S., Vartanian J.P. (2005). Extensive editing of both hepatitis B virus DNA strands by APOBEC3 cytidine deaminases *in vitro* and *in vivo*. Proc. Natl. Acad. Sci. U.S.A..

[259] Fehrholz M., Kendl S., Prifert C. (2012). The innate antiviral factor APOBEC3G targets replication of measles, mumps and respiratory syncytial viruses. J. Gen. Virol..

[260] Hrecka K., Hao C., Gierszewska M. (2011). Vpx relieves inhibition of HIV-1 infection of macrophages mediated by the SAMHD1 protein. Nature.

[261] Laguette N., Sobhian B., Casartelli N. (2011). SAMHD1 is the dendritic- and myeloid-cell-specific HIV-1 restriction factor counteracted by Vpx. Nature.

[262] Goldstone D.C., Ennis-Adeniran V., Hedden J.J. (2011). HIV-1 restriction factor SAMHD1 is a deoxynucleoside triphosphate triphosphohydrolase. Nature.

[263] Powell R.D., Holland P.J., Hollis T., Perrino F.W. (2011). Aicardi-Goutieres syndrome gene and HIV-1 restriction factor SAMHD1 is a dGTP-regulated deoxynucleotide triphosphohydrolase. J. Biol. Chem..

[264] Sayah D.M., Sokolskaja E., Berthoux L., Luban J. (2004). Cyclophilin A retrotransposition into TRIM5 explains owl monkey resistance to HIV-1. Nature.

[265] Roa A., Hayashi F., Yang Y. (2012). RING domain mutations uncouple TRIM5alpha restriction of HIV-1 from inhibition of reverse transcription and acceleration of uncoating. J. Virol..

[266] Liu F.L., Qiu Y.Q., Li H. (2011). An HIV-1 resistance polymorphism in TRIM5alpha gene among Chinese intravenous drug users. J. Acquir. Immune. Defic. Syndr..

[267] Price H., Lacap P., Tuff J. (2010). A TRIM5alpha exon 2 polymorphism is associated with protection from HIV-1 infection in the Pumwani sex worker cohort. AIDS.

[268] Javanbakht H., An P., Gold B. (2006). Effects of human TRIM5alpha polymorphisms on antiretroviral function and susceptibility to human immunodeficiency virus infection. Virology.

[269] Le T.A., Willey S., Neil S.J. (2011). Antiviral inhibition of enveloped virus release by tetherin/BST-2: Action and counteraction. Viruses.

[270] Kuhl B.D., Cheng V., Wainberg M.A., Liang C. (2011). Tetherin and its viral antagonists. J. Neuroimmune. Pharmacol..

[271] Bego M.G., Mercier J., Cohen E.A. (2012). Virus-activated interferon regulatory factor 7 upregulates expression of the interferon-regulated BST2 gene independently of interferon signaling. J. Virol..

[272] Jouvenet N., Neil S.J., Zhadina M. (2009). Broad-spectrum inhibition of retroviral and filoviral particle release by tetherin. J. Virol..

[273] Barrett B.S., Smith D.S., Li S.X., Guo K., Hasenkrug K.J., Santiago M.L. (2012). A single nucleotide polymorphism in tetherin promotes retrovirus restriction *in vivo*. PLoS Pathog.

[274] Kuhl B.D., Sloan R.D., Donahue D.A., Bar-Magen T., Liang C., Wainberg M.A. (2010). Tetherin restricts direct cell-to-cell infection of HIV-1. Retrovirology.

[275] Dafa-Berger A., Kuzmina A., Fassler M., Yitzhak-Asraf H., Shemer-Avni Y., Taube R. (2012). Modulation of hepatitis C virus release by the interferon-induced protein BST-2/tetherin. Virology.

[276] Mansouri M., Viswanathan K., Douglas J.L. (2009). Molecular mechanism of BST2/tetherin downregulation by K5/MIR2 of Kaposi's sarcoma-associated herpesvirus. J. Virol..

[277] Yasuda J. (2012). Ebolavirus Replication and Tetherin/BST-2. Front Microbiol..

[278] Mangeat B., Cavagliotti L., Lehmann M. (2012). Influenza Virus Partially Counteracts Restriction Imposed by Tetherin/BST-2. J. Biol. Chem..

[279] Viswanathan K., Smith M.S., Malouli D., Mansouri M., Nelson J.A., Fruh K. (2011). BST2/Tetherin enhances entry of human cytomegalovirus. PLoS Pathog.

[280] Weidner J.M., Jiang D., Pan X.B., Chang J., Block T.M., Guo J.T. (2010). Interferon-induced cell membrane proteins, IFITM3 and tetherin, inhibit vesicular stomatitis virus infection via distinct mechanisms. J. Virol..

[281] Perez-Caballero D., Zang T., Ebrahimi A. (2009). Tetherin inhibits HIV-1 release by directly tethering virions to cells. Cell.

[282] Tissot C., Mechti N. (1995). Molecular cloning of a new interferon-induced factor that represses human immunodeficiency virus type 1 long terminal repeat expression. J. Biol. Chem..

[283] Bouazzaoui A., Kreutz M., Eisert V. (2006). Stimulated trans-acting factor of 50 kDa (Staf50) inhibits HIV-1 replication in human monocyte-derived macrophages. Virology.

[284] Kajaste-Rudnitski A., Marelli S.S., Pultrone C. (2011). TRIM22 inhibits HIV-1 transcription independently of its E3 ubiquitin ligase activity, Tat, and NF-kappaB-responsive long terminal repeat elements. J. Virol..

[285] Barr S.D., Smiley J.R., Bushman F.D. (2008). The interferon response inhibits HIV particle production by induction of TRIM22. PLoS Pathog.

[286] Gao B., Duan Z., Xu W., Xiong S. (2009). Tripartite motif-containing 22 inhibits the activity of hepatitis B virus core promoter, which is dependent on nuclear-located RING domain. Hepatology.

[287] Eldin P., Papon L., Oteiza A., Brocchi E., Lawson T.G., Mechti N. (2009). TRIM22 E3 ubiquitin ligase activity is required to mediate antiviral activity against encephalomyocarditis virus. J. Gen. Virol..

[288] Samuel C.E. (2011). Adenosine deaminases acting on RNA (ADARs) are both antiviral and proviral. Virology.

[289] Biswas N., Wang T., Ding M. (2012). ADAR1 is a novel multi targeted anti-HIV-1 cellular protein. Virology.

[290] Taylor D.R., Puig M., Darnell M.E., Mihalik K., Feinstone S.M. (2005). New antiviral pathway that mediates hepatitis C virus replicon interferon sensitivity through ADAR1. J. Virol..

[291] Jayan G.C., Casey J.L. (2002). Inhibition of hepatitis delta virus RNA editing by short inhibitory RNA-mediated knockdown of ADAR1 but not ADAR2 expression. J. Virol..

[292] Wong S.K., Lazinski D.W. (2002). Replicating hepatitis delta virus RNA is edited in the nucleus by the small form of ADAR1. Proc. Natl. Acad. Sci. U.S.A..

[293] Hartwig D., Schutte C., Warnecke J. (2006). The large form of ADAR 1 is responsible for enhanced hepatitis delta virus RNA editing in interferon-alpha-stimulated host cells. J. Viral. Hepat..

[294] Doria M., Tomaselli S., Neri F. (2011). ADAR2 editing enzyme is a novel human immunodeficiency virus-1 proviral factor. J. Gen. Virol..

[295] Clerzius G., Gelinas J.F., Daher A., Bonnet M., Meurs E.F., Gatignol A. (2009). ADAR1 interacts with PKR during human immunodeficiency virus infection of lymphocytes and contributes to viral replication. J. Virol..

[296] Phuphuakrat A., Kraiwong R., Boonarkart C., Lauhakirti D., Lee T.H., Auewarakul P. (2008). Double-stranded RNA adenosine deaminases enhance expression of human immunodeficiency virus type 1 proteins. J. Virol..

[297] Nie Y., Hammond G.L., Yang J.H. (2007). Double-stranded RNA deaminase ADAR1 increases host susceptibility to virus infection. J. Virol..

[298] Li Z., Wolff K.C., Samuel C.E. (2010). RNA adenosine deaminase ADAR1 deficiency leads to increased activation of protein kinase PKR and reduced vesicular stomatitis virus growth following interferon treatment. Virology.

[299] Li Z., Okonski K.M., Samuel C.E. (2012). Adenosine deaminase acting on RNA 1 (ADAR1) suppresses the induction of interferon by measles virus. J. Virol..

[300] Toth A.M., Li Z., Cattaneo R., Samuel C.E. (2009). RNA-specific adenosine deaminase ADAR1 suppresses measles virus-induced apoptosis and activation of protein kinase PKR. J. Biol. Chem..

[301] Rose W.A., McGowin C.L., Pyles R.B. (2009). FSL-1, a bacterial-derived toll-like receptor 2/6 agonist, enhances resistance to experimental HSV-2 infection. Virol. J..

[302] Gill N., Deacon P.M., Lichty B., Mossman K.L., Ashkar A.A. (2006). Induction of innate immunity against herpes simplex virus type 2 infection via local delivery of Toll-like receptor ligands correlates with beta interferon production. J. Virol..

[303] Thibault S., Tardif M.R., Barat C., Tremblay M.J. (2007). TLR2 signaling renders quiescent naive and memory CD4+ T cells more susceptible to productive infection with X4 and R5 HIV-type 1. J. Immunol..

[304] de Jong M.A., de Witte L., Oudhoff M.J., Gringhuis S.I., Gallay P., Geijtenbeek T.B. (2008). TNF-alpha and TLR agonists increase susceptibility to HIV-1 transmission by human Langerhans cells *ex vivo*. J. Clin. Invest..

[305] Thibault S., Fromentin R., Tardif M.R., Tremblay M.J. (2009). TLR2 and TLR4 triggering exerts contrasting effects with regard to HIV-1 infection of human dendritic cells and subsequent virus transfer to CD4+ T cells. Retrovirology.

[306] Datta S.K., Redecke V., Prilliman K.R. (2003). A subset of Toll-like receptor ligands induces cross-presentation by bone marrow-derived dendritic cells. J. Immunol..

[307] Weck M.M., Grunebach F., Werth D., Sinzger C., Bringmann A., Brossart P. (2007). TLR ligands differentially affect uptake and presentation of cellular antigens. Blood.

[308] Suh H.S., Zhao M.L., Choi N., Belbin T.J., Brosnan C.F., Lee S.C. (2009). TLR3 and TLR4 are innate antiviral immune receptors in human microglia: Role of IRF3 in modulating antiviral and inflammatory response in the CNS. Virology.

[309] Rivieccio M.A., Suh H.S., Zhao Y. (2006). TLR3 Ligation Activates an Antiviral Response in Human Fetal Astrocytes: A Role for Viperin/cig5. J. Immunol..

[310] Brichacek B., Vanpouille C., Kiselyeva Y. (2010). Contrasting roles for TLR ligands in HIV-1 pathogenesis. PLoS ONE.

[311] Herbst-Kralovetz M.M., Pyles R.B. (2006). Quantification of poly(I:C)-mediated protection against genital herpes simplex virus type 2 infection. J. Virol..

[312] Nazli A., Yao X.D., Smieja M., Rosenthal K.L., Ashkar A.A., Kaushic C. (2009). Differential induction of innate anti-viral responses by TLR ligands against Herpes simplex virus, type 2, infection in primary genital epithelium of women. Antiviral. Res..

[313] MacDonald E.M., Savoy A., Gillgrass A. (2007). Susceptibility of human female primary genital epithelial cells to herpes simplex virus, type-2 and the effect of TLR3 ligand and sex hormones on infection. Biol. Reprod..

[314] Ashkar A.A., Yao X.D., Gill N., Sajic D., Patrick A.J., Rosenthal K.L. (2004). Toll-like receptor (TLR)-3, but not TLR4, agonist protects against genital herpes infection in the absence of inflammation seen with CpG DNA. J. Infect. Dis..

[315] Boivin N., Sergerie Y., Rivest S., Boivin G. (2008). Effect of pretreatment with toll-like receptor agonists in a mouse model of herpes simplex virus type 1 encephalitis. J. Infect. Dis..

[316] Lai Y., Adhikarakunnathu S. (2011). LL37 and cationic peptides enhance TLR3 signaling by viral double-stranded RNAs. PLoS ONE.

[317] Lai Y., Yi G., Chen A. (2011). Viral double-strand RNA-binding proteins can enhance innate immune signaling by toll-like Receptor 3. PLoS ONE.

[318] Funderburg N., Luciano A.A., Jiang W., Rodriguez B., Sieg S.F., Lederman M.M. (2008). Toll-like receptor ligands induce human T cell activation and death, a model for HIV pathogenesis. PLoS ONE.

[319] Warger T., Osterloh P., Rechtsteiner G. (2006). Synergistic activation of dendritic cells by combined Toll-like receptor ligation induces superior CTL responses *in vivo*. Blood.

[320] Napolitani G., Rinaldi A., Bertoni F., Sallusto F., Lanzavecchia A. (2005). Selected Toll-like receptor agonist combinations synergistically trigger a T helper type 1-polarizing program in dendritic cells. Nat. Immunol..

[321] Kanzler H., Barrat F.J., Hessel E.M., Coffman R.L. (2007). Therapeutic targeting of innate immunity with Toll-like receptor agonists and antagonists. Nat. Med..

[322] Gil-Torregrosa B.C., Lennon-Dumenil A.M., Kessler B. (2004). Control of cross-presentation during dendritic cell maturation. Eur. J. Immunol..

[323] Wilson N.S., Behrens G.M., Lundie R.J. (2006). Systemic activation of dendritic cells by Toll-like receptor ligands or malaria infection impairs cross-presentation and antiviral immunity. Nat. Immunol..

[324] Ashkar A.A., Mossman K.L., Coombes B.K., Gyles C.L., Mackenzie R. (2008). FimH adhesin of type 1 fimbriae is a potent inducer of innate antimicrobial responses which requires TLR4 and type 1 interferon signalling. PLoS Pathog.

[325] Equils O., Salehi K.K., Cornataeanu R. (2006). Repeated lipopolysaccharide (LPS) exposure inhibits HIV replication in primary human macrophages. Microbes. Infect..

[326] Equils O., Faure E., Thomas L., Bulut Y., Trushin S., Arditi M. (2001). Bacterial lipopolysaccharide activates HIV long terminal repeat through Toll-like receptor 4. J. Immunol..

[327] Berg R.S., Aggerholm A., Bertelsen L.S., Ostergaard L., Paludan S.R. (2009). Role of mitogen-activated protein kinases, nuclear factor-kappaB, and interferon regulatory factor 3 in Toll-like receptor 4-mediated activation of HIV long terminal repeat. APMIS.

[328] Brenchley J.M., Price D.A., Schacker T.W. (2006). Microbial translocation is a cause of systemic immune activation in chronic HIV infection. Nat. Med..

[329] Hemmi H., Kaisho T., Takeuchi O. (2002). Small anti-viral compounds activate immune cells via the TLR7 MyD88-dependent signaling pathway. Nat. Immunol..

[330] Wagner T.L., Ahonen C.L., Couture A.M. (1999). Modulation of TH1 and TH2 cytokine production with the immune response modifiers, R-848 and imiquimod. Cell Immunol..

[331] Lore K., Betts M.R., Brenchley J.M. (2003). Toll-like receptor ligands modulate dendritic cells to augment cytomegalovirus- and HIV-1-specific T cell responses. J. Immunol..

[332] Prins R.M., Craft N., Bruhn K.W. (2006). The TLR-7 agonist, imiquimod, enhances dendritic cell survival and promotes tumor antigen-specific T cell priming: relation to central nervous system antitumor immunity. J. Immunol..

[333] Oh J.Z., Kurche J.S., Burchill M.A., Kedl R.M. (2011). TLR7 enables cross-presentation by multiple dendritic cell subsets through a type I IFN-dependent pathway. Blood.

[334] Hart O.M., Athie-Morales V., O'connor G.M., Gardiner C.M. (2005). TLR7/8-Mediated Activation of Human NK Cells Results in Accessory Cell-Dependent IFN-{gamma} Production. J. Immunol..

[335] Ablasser A., Poeck H., Anz D. (2009). Selection of molecular structure and delivery of RNA oligonucleotides to activate TLR7 versus TLR8 and to induce high amounts of IL-12p70 in primary human monocytes. J. Immunol..

[336] Mark K.E., Corey L., Meng T.C. (2007). Topical resiquimod 0.01% gel decreases herpes simplex virus type 2 genital shedding: A randomized, controlled trial. J. Infect. Dis..

[337] McCluskie M.J., Cartier J.L., Patrick A.J. (2006). Treatment of intravaginal HSV-2 infection in mice: A comparison of CpG oligodeoxynucleotides and resiquimod (R-848). Antiviral Res..

[338] Hammerbeck D.M., Burleson G.R., Schuller C.J. (2007). Administration of a dual toll-like receptor 7 and toll-like receptor 8 agonist protects against influenza in rats. Antiviral Res..

[339] Wille-Reece U., Flynn B.J., Lore K. (2006). Toll-like receptor agonists influence the magnitude and quality of memory T cell responses after prime-boost immunization in nonhuman primates. J. Exp. Med..

[340] Wille-Reece U., Wu C.Y., Flynn B.J., Kedl R.M., Seder R.A. (2005). Immunization with HIV-1 Gag protein conjugated to a TLR7/8 agonist results in the generation of HIV-1 Gag-specific Th1 and CD8+ T cell responses. J. Immunol..

[341] Velasquez L.S., Hjelm B.E., Arntzen C.J., Herbst-Kralovetz M.M. (2010). An intranasally delivered Toll-like receptor 7 agonist elicits robust systemic and mucosal responses to Norwalk virus-like particles. Clin. Vaccine Immunol..

[342] Weeratna R.D., Makinen S.R., McCluskie M.J., Davis H.L. (2005). TLR agonists as vaccine adjuvants: comparison of CpG ODN and Resiquimod (R-848). Vaccine.

[343] Haasnoot J., Westerhout E.M., Berkhout B. (2007). RNA interference against viruses: Strike and counterstrike. Nat. Biotechnol..

[344] Khairuddin N., Gantier M.P., Blake S.J. (2011). siRNA-induced immunostimulation through TLR7 promotes antitumoral activity against HPV-driven tumors *in vivo*. Immunol. Cell Biol..

[345] Mureith M.W., Chang J.J., Lifson J.D., Ndung'u T., Altfeld M. (2010). Exposure to HIV-1-encoded Toll-like receptor 8 ligands enhances monocyte response to microbial encoded Toll-like receptor 2/4 ligands. AIDS.

[346] Bukh A.R., Melchjorsen J., Offersen R. (2011). Endotoxemia is associated with altered innate and adaptive immune responses in untreated HIV-1 infected individuals. PLoS ONE.

[347] Krieg A.M. (2006). Therapeutic potential of Toll-like receptor 9 activation. Nat. Rev. Drug. Discov..

[348] Kerkmann M., Rothenfusser S., Hornung V. (2003). Activation with CpG-A and CpG-B oligonucleotides reveals two distinct regulatory pathways of type I IFN synthesis in human plasmacytoid dendritic cells. J. Immunol..

[349] Becker Y. (2005). CpG ODNs treatments of HIV-1 infected patients may cause the decline of transmission in high risk populations - a review, hypothesis and implications. Virus Genes.

[350] Gallichan W.S., Woolstencroft R.N., Guarasci T., McCluskie M.J., Davis H.L., Rosenthal K.L. (2001). Intranasal immunization with CpG oligodeoxynucleotides as an adjuvant dramatically increases IgA and protection against herpes simplex virus-2 in the genital tract. J. Immunol..

[351] Sajic D., Ashkar A.A., Patrick A.J. (2003). Parameters of CpG oligodeoxynucleotide-induced protection against intravaginal HSV-2 challenge. J. Med. Virol..

[352] Harandi A.M. (2004). The potential of immunostimulatory CpG DNA for inducing immunity against genital herpes: opportunities and challenges. J. Clin. Virol..

[353] Equils O., Schito M.L., Karahashi H. (2003). Toll-like receptor 2 (TLR2) and TLR9 signaling results in HIV-long terminal repeat trans-activation and HIV replication in HIV-1 transgenic mouse spleen cells: Implications of simultaneous activation of TLRs on HIV replication. J. Immunol..

[354] Jensen K.M., Melchjorsen J., Dagnaes-Hansen F. (2012). Timing of toll-like receptor 9 agonist administration in pneumococcal vaccination impact both humoral and cellular immune responses as well as nasopharyngeal colonization in mice. Infect. Immun..

[355] Pichlmair A., Diebold S.S., Gschmeissner S. (2007). Tubulovesicular structures within vesicular stomatitis virus G protein-pseudotyped lentiviral vector preparations carry DNA and stimulate antiviral responses via Toll-like receptor 9. J. Virol..

[356] Zhu J., Huang X., Yang Y. (2007). Innate immune response to adenoviral vectors is mediated by both Toll-like receptor-dependent and -independent pathways. J. Virol..

[357] Coulombe F., Fiola S., Akira S., Cormier Y., Gosselin J. (2012). Muramyl dipeptide induces NOD2-dependent Ly6C(high) monocyte recruitment to the lungs and protects against influenza virus infection. PLoS ONE.

[358] Shafique M., Wilschut J., de H.A. (2012). Induction of mucosal and systemic immunity against respiratory syncytial virus by inactivated virus supplemented with TLR9 and NOD2 ligands. Vaccine.

[359] Zaks K., Jordan M., Guth A. (2006). Efficient immunization and cross-priming by vaccine adjuvants containing TLR3 or TLR9 agonists complexed to cationic liposomes. J. Immunol..

[360] Bernstein D.I., Cardin R.D., Bravo F.J. (2009). Potent adjuvant activity of cationic liposome-DNA complexes for genital herpes vaccines. Clin. Vaccine Immunol..

[361] Bernstein D.I., Farley N., Bravo F.J. (2010). The adjuvant CLDC increases protection of a herpes simplex type 2 glycoprotein D vaccine in guinea pigs. Vaccine.

[362] Delaloye J., Roger T., Steiner-Tardivel Q.G. (2009). Innate immune sensing of modified vaccinia virus Ankara (MVA) is mediated by TLR2-TLR6, MDA-5 and the NALP3 inflammasome. PLoS Pathog.

[363] Lladser A., Mougiakakos D., Tufvesson H. (2011). DAI (DLM-1/ZBP1) as a genetic adjuvant for DNA vaccines that promotes effective antitumor CTL immunity. Mol. Ther..

[364] Ishii K.J., Kawagoe T., Koyama S. (2008). TANK-binding kinase-1 delineates innate and adaptive immune responses to DNA vaccines. Nature.

[365] Huang L., Lemos H.P., Li L. (2012). Engineering DNA nanoparticles as immunomodulatory teagents that activate regulatory T cells. J. Immunol..

[366] Equils O., Shapiro A., Madak Z., Liu C., Lu D. (2004). Human immunodeficiency virus type 1 protease inhibitors block toll-like receptor 2 (TLR2)- and TLR4-Induced NF-kappaB activation. Antimicrob. Agents. Chemother..

[367] Wallet M.A., Reist C.M., Williams J.C. (2012). The HIV-1 protease inhibitor nelfinavir activates PP2 and inhibits MAPK signaling in macrophages: A pathway to reduce inflammation. J. Leukoc. Biol..

[368] Danaher R.J., Kaetzel C.S., Greenberg R.N., Wang C., Bruno M.E., Miller C.S. (2010). HIV protease inhibitors alter innate immune response signaling to double-stranded RNA in oral epithelial cells: Implications for immune reconstitution inflammatory syndrome?. AIDS.

[369] Pajonk F., Himmelsbach J., Riess K., Sommer A., McBride W.H. (2002). The human immunodeficiency virus (HIV)-1 protease inhibitor saquinavir inhibits proteasome function and causes apoptosis and radiosensitization in non-HIV-associated human cancer cells. Cancer Res..

[370] Kurata S. (1994). Potential of azidothymidine to activate the HIV-1 promoter. J. Biol. Chem..

[371] Melchjorsen J., Risør M.W., Sogaard O.S. (2011). Tenofovir selectively regulates production of inflammatory cytokines and shifts the IL-12 / IL-10 balance in human primary cells. J. Acquir. Immune. Defic. Syndr..

[372] Kurokawa M., Ghosh S.K., Ramos J.C. (2005). Azidothymidine inhibits NF-kappaB and induces Epstein-Barr virus gene expression in Burkitt lymphoma. Blood.

[373] Martin A.M., Almeida C.A., Cameron P. (2007). Immune responses to abacavir in antigen-presenting cells from hypersensitive patients. AIDS.

[374] Van Rompay K.K., Marthas M.L., Bischofberger N. (2004). Tenofovir primes rhesus macaque cells *in vitro* for enhanced interleukin-12 secretion. Antiviral Res..

[375] Zidek Z., Frankova D., Holy A. (2001). Activation by 9-(R)-[2-(phosphonomethoxy)propyl]adenine of chemokine (RANTES, macrophage inflammatory protein 1alpha) and cytokine (tumor necrosis factor alpha, interleukin-10 [IL-10], IL-1beta) production. Antimicrob. Agents. Chemother..

[376] Zidek Z., Kmonickova E., Holy A. (2007). Secretion of antiretroviral chemokines by human cells cultured with acyclic nucleoside phosphonates. Eur. J. Pharmacol..

[377] Abdool K.Q., Abdool Karim S.S., Frohlich J.A. (2010). Effectiveness and safety of tenofovir gel, an antiretroviral microbicide, for the prevention of HIV infection in women. Science.

[378] (2010). FHI and the Centre for the AIDS Programme of Research in South Africa; Factsheet: CAPRISA 004 Trial and the impact of tenofovir gel on herpes simplex virus type-2 infections.

[379] Mesquita P.M., Rastogi R., Segarra T.J. (2012). Intravaginal ring delivery of tenofovir disoproxil fumarate for prevention of HIV and herpes simplex virus infection. J. Antimicrob. Chemother..

[380] Andrei G., Lisco A., Vanpouille C. (2011). Topical tenofovir, a microbicide effective against HIV, inhibits herpes simplex virus-2 replication. Cell Host Microbe.

[381] Vibholm L., Reinert L.S., Sogaard O.S. (2012). Antiviral and immunological effects of tenofovir microbicide in vaginal herpes simplex virus 2 infection. AIDS Res. Hum. Retroviruses.

[382] Kikuchi T., Hagiwara K., Honda Y. (2002). Clarithromycin suppresses lipopolysaccharide-induced interleukin-8 production by human monocytes through AP-1 and NF-kappa B transcription factors. J. Antimicrob. Chemother..

[383] Khair O.A., Devalia J.L., Abdelaziz M.M., Sapsford R.J., Davies R.J. (1995). Effect of erythromycin on Haemophilus influenzae endotoxin-induced release of IL-6, IL-8 and sICAM-1 by cultured human bronchial epithelial cells. Eur. Respir. J..

[384] Schultz M.J., Speelman P., Van Der Poll T. (2001). Erythromycin inhibits Pseudomonas aeruginosa-induced tumour necrosis factor-alpha production in human whole blood. J. Antimicrob. Chemother..

[385] Li D.Q., Zhou N., Zhang L., Ma P., Pflugfelder S.C. (2010). Suppressive effects of azithromycin on zymosan-induced production of proinflammatory mediators by human corneal epithelial cells. Invest. Ophthalmol. Vis. Sci..

[386] Murphy D.M., Forrest I.A., Corris P.A. (2008). Azithromycin attenuates effects of lipopolysaccharide on lung allograft bronchial epithelial cells. J. Heart Lung Transplant..

[387] Morikawa K., Zhang J., Nonaka M., Morikawa S. (2002). Modulatory effect of macrolide antibiotics on the Th1- and Th2-type cytokine production. Int. J. Antimicrob. Agents..

[388] Murphy B.S., Sundareshan V., Cory T.J., Hayes D., Anstead M.I., Feola D.J. (2008). Azithromycin alters macrophage phenotype. J. Antimicrob. Chemother..

[389] Darisipudi M.N., Allam R., Rupanagudi K.V., Anders H.J. (2011). Polyene Macrolide Antifungal Drugs Trigger Interleukin-1beta Secretion by Activating the NLRP3 Inflammasome. PLoS ONE.

[390] Razonable R.R., Henault M., Watson H.L., Paya C.V. (2005). Nystatin induces secretion of interleukin (IL)-1beta, IL-8, and tumor necrosis factor alpha by a toll-like receptor-dependent mechanism. Antimicrob. Agents. Chemother..

[391] Razonable R.R., Henault M., Lee L.N. (2005). Secretion of proinflammatory cytokines and chemokines during amphotericin B exposure is mediated by coactivation of toll-like receptors 1 and 2. Antimicrob. Agents. Chemother..

[392] Rizzo A., Paolillo R., Guida L., Annunziata M., Bevilacqua N., Tufano M.A. (2010). Effect of metronidazole and modulation of cytokine production on human periodontal ligament cells. Int. Immunopharmacol..

[393] Hirata N., Hiramatsu K., Kishi K., Yamasaki T., Ichimiya T., Nasu M. (2001). Pretreatment of mice with clindamycin improves survival of endotoxic shock by modulating the release of inflammatory cytokines. Antimicrob. Agents. Chemother..

[394] Nakano T., Hiramatsu K., Kishi K., Hirata N., Kadota J., Nasu M. (2003). Clindamycin modulates inflammatory-cytokine induction in lipopolysaccharide-stimulated mouse peritoneal macrophages. Antimicrob. Agents. Chemother..

[395] Araujo F.G., Slifer T.L., Remington J.S. (2002). Inhibition of secretion of interleukin-1alpha and tumor necrosis factor alpha by the ketolide antibiotic telithromycin. Antimicrob. Agents. Chemother..

[396] Sugiura Y., Hiramatsu K., Hamauzu R. (2012). Mitogen-activated protein kinases-dependent induction of hepatocyte growth factor production in human dermal fibroblasts by the antibiotic polymyxin B. Cytokine.

[397] Ichinohe T., Pang I.K., Kumamoto Y. (2011). Microbiota regulates immune defense against respiratory tract influenza A virus infection. Proc. Natl. Acad. Sci. U.S.A..

